# Discovery of Partial Differential Equations from Highly Noisy and Sparse Data with Physics-Informed Information Criterion

**DOI:** 10.34133/research.0147

**Published:** 2023-05-19

**Authors:** Hao Xu, Junsheng Zeng, Dongxiao Zhang

**Affiliations:** ^1^ BIC-ESAT, ERE, and SKLTCS, College of Engineering, Peking University, Beijing 100871, P. R. China.; ^2^ Institute of Applied Physics and Computational Mathematics, Beijing 100088, P. R. China.; ^3^ Eastern Institute for Advanced Study, Eastern Institute of Technology, Ningbo 315200, Zhejiang, P. R. China.; ^4^ National Center for Applied Mathematics Shenzhen (NCAMS), Southern University of Science and Technology, Shenzhen 518055, Guangdong, P. R. China.; ^5^ Department of Mathematics and Theories, Peng Cheng Laboratory, Shenzhen 518000, Guangdong, P. R. China.

## Abstract

Data-driven discovery of partial differential equations (PDEs) has recently made tremendous progress, and many canonical PDEs have been discovered successfully for proof of concept. However, determining the most proper PDE without prior references remains challenging in terms of practical applications. In this work, a physics-informed information criterion (PIC) is proposed to measure the parsimony and precision of the discovered PDE synthetically. The proposed PIC achieves satisfactory robustness to highly noisy and sparse data on 7 canonical PDEs from different physical scenes, which confirms its ability to handle difficult situations. The PIC is also employed to discover unrevealed macroscale governing equations from microscopic simulation data in an actual physical scene. The results show that the discovered macroscale PDE is precise and parsimonious and satisfies underlying symmetries, which facilitates understanding and simulation of the physical process. The proposition of the PIC enables practical applications of PDE discovery in discovering unrevealed governing equations in broader physical scenes.

## Introduction

With the development of machine learning techniques and computational power, the data-driven discovery of partial differential equations (PDEs) has made tremendous progress over the past years. Unlike the paradigm of deriving physical laws from first principles or constitution laws, it provides an alternative path by directly discovering governing equations from observation data, which is more appropriate and practical for systems with elusive underlying mechanisms.

In essence, the discovery of PDEs constitutes a sparse regression task in which a few terms with nonzero coefficients are identified from unlimited candidates. Similar to model selection, criterion and optimization methods play an important role in the field of PDE discovery. In the early stage of research, sparse regression methods, including Lasso [1], sequential threshold ridge regression (STRidge) [2] and SINDy [3], were adopted to discover PDEs from a limited candidate library given beforehand. These sparse regression-based methods essentially utilize regularization terms (e.g., *L*_1_-norm or *L*_2_-norm) to conduct continuous optimization by eliminating terms with tiny coefficients, which has high calculation efficiency but requires a predetermined complete candidate library. Therefore, genetic algorithm-based methods were developed to discover PDEs from unlimited potential combinations generated from several basic genes by cross-over and mutation [4,5]. Different from sparse regression, the genetic algorithm adopts random search optimization to evolve the best PDE, which is more flexible. Utilization of the *L*_0_-norm in the fitness is employed as the criterion to select the optimal combinations. The incorporation of symbolic regression [6] further relaxes the restrictions of the candidate library and makes the discovery of free-form PDEs possible. Subsequent works [7–12] mainly focused on the incorporation of diversified techniques to facilitate the optimization process. However, reformation of criteria that can bring essential improvements is currently absent in the literature.

The rapid development of deep learning techniques has brought additional possibilities to PDE discovery. Automatic differentiation of the neural network has greatly improved the flexibility and accuracy of derivatives calculation, which assists in solving the problem of sparse data and high noise [13–15]. In addition to the neural network, auxiliary techniques, such as the integral form [16], have been incorporated to reduce the impact of data noise and enhance the accuracy of data analysis. Among neural network-based techniques, the physics-informed neural network (PINN) [17] shows excellent robustness and accuracy when identifying PDEs from sparse and noisy data [18–20]. The basic concept of these works can be summarized as alternative direction optimization [20], which sequentially optimizes both the model and the discovered governing equation through PINN. However, the optimization cycle sometimes cannot converge, especially when faced with extremely high noise or complex physical processes. This is because the performance of alternative direction optimization relies on a benign optimization cycle, which means that a deficient initial model may lead to a deviated physical constraint that degrades model performance.

Moreover, the present methods mentioned above largely remain in the stage of proof of concept, where the data originated from the numerical solution of canonical PDEs or systems. In terms of practical applications, several challenges will emerge, including sparse and noisy data, stability, unavailable referenced PDE, and interpretability. Specifically, the outcomes of existing methods vary with the selection of core hyperparameters that control the magnitude of regularization and thresholds, which is unfavorable in practical applications since the hyperparameters are difficult to adjust without a referenced PDE. Meanwhile, current criteria and optimization approaches are insufficient to obtain a physically interpretable governing equation that can balance precision and parsimony well in practical scenes. This problem has been revealed in our prior work [21], which took a tentative step to adopt an existing method to discover unrevealed governing equations in the field of proppant transport. It was discovered that although the governing equation discovered by the existing method has small error, some redundant terms seriously hinder the subsequent numerical solution and understanding of physical meaning.

In general, a proper PDE for describing a physical process should satisfy 3 principles: precision; parsimony; and interpretability. Inspired by these principles, an entirely new information criterion, called the physics-informed information criterion (PIC), is proposed in this work, which combines the measurement of precision and parsimony to select a proper PDE with physical interpretability. In the PIC, the moving horizon technique is introduced to identify redundant terms and measure parsimony. At the same time, the PINN method is employed to estimate precision without solving potential PDEs numerically. Experiments in this work have confirmed the ability of the proposed PIC to discover the most proper PDE in both proof-of-concept and practical scenes. Sufficient comparison with conventional information criterion, classic sparse regression-based methods, and current PINN-based methods further confirms the superiority of the PIC to existing methods.

The contributions of this work can be summarized as follows:

1. A PDE discovery framework with a new criterion. The proposed PIC provides a new yet efficient criterion to select the optimal PDE from potential combinations. This means that the PIC does not involve a sophisticated optimization cycle to gradually drop tiny-coefficient terms or adjust physical constraints, which leads to better stability and accuracy.

2. High robustness to sparse and highly noisy data. For 7 canonical PDEs from different physical fields, satisfactory outcomes are obtained by the PIC for these PDEs with sparse and highly noisy data. In some cases, the PIC is robust up to 200% Gaussian noise.

3. High flexibility and stability to hyperparameters of penalty term. The proposed PIC method offers greater flexibility in the selection of hyperparameters of the penalty term, which remains stable even in the presence of high levels of noise. This feature enhances the method’s suitability for broader tasks.

4. Practical application. The PIC can discover the most proper PDE in practical applications where the referenced PDE has not been revealed previously. The discovered PDE is parsimonious, accurate, and even satisfies symmetry, which facilitates physical understanding of the underlying processes.

## Results

### Overview of the PIC

In this work, the mathematical form of the PDE discovery problem is written as follows:

$$U_{t}=\Omega (u,u_{x},{\mathrm{uu}}_{x},u^{2}u_{x},u_{\mathrm{xx}},\ldots )\cdot \vec{\xi },$$ (1)

where *U_t_* is the left-hand side (LHS) term, which can be the first- or second-order derivative of time; Ω is the linear combination operator; and $\vec{\xi }$ is the coefficient vector. For PDE discovery, it aims to identify several terms with nonzero coefficients from unlimited combinations of *u* and its spatial derivatives.

**Fig. 1.**
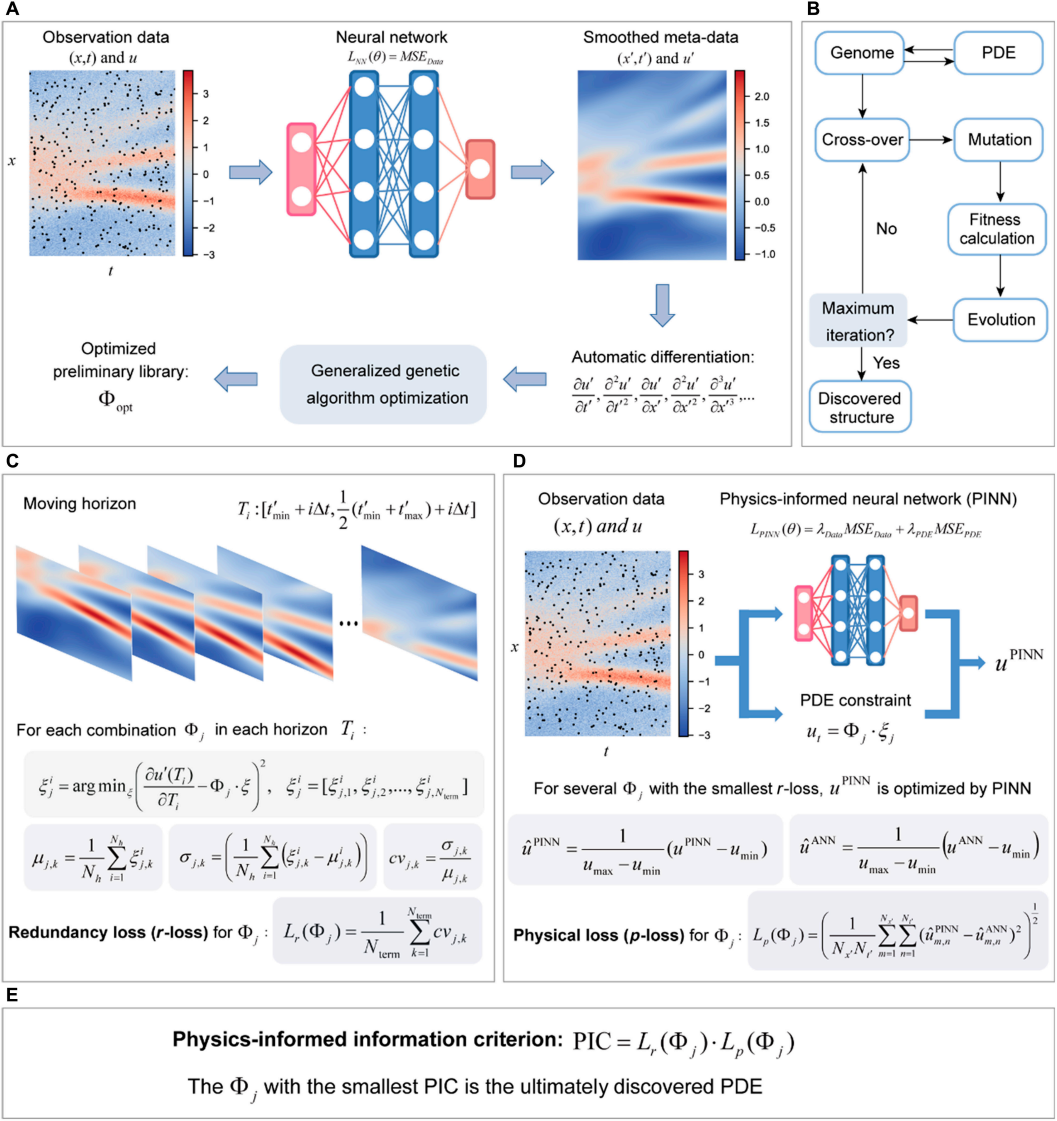
Overview of the PIC. (A) The flow chart of training the ANN, calculating derivatives via automatic differentiation, and obtaining the optimized preliminary library. (B) The flow chart of the generalized genetic algorithm optimization. (C) The process of calculating *r*-loss for each possible combination by the moving horizon technique. Here, *N_h_* is the number of moving horizons, and *N*_term_ is the number of terms in the combination Φ*_j_*. (D) The process of calculating the *p*-loss by training PINN with potential PDEs as the physical constraints. (E) The definition of the PIC.

An overview of the PIC is provided in Fig. 1. An artificial neural network (ANN) is utilized here to construct a surrogate model to generate smoothed meta-data and calculate derivatives. Considering that solving Eq. 1 is a non-deterministic polynomial-time (NP)-hard problem with unlimited combinations, the generalized genetic algorithm optimization (illustrated in Fig. 1B, detailed in The generalized genetic algorithm optimization in Materials and Methods) is employed to select an optimized preliminary library Φ_opt_ from unlimited combinations and convert the problem to a finite-dimensional problem (Fig. 1A). For the optimized preliminary library with a few terms, the possibilities of the combinations are countable, and thus, it is feasible to measure the PIC of each combination Φ*_j_* and select the most proper PDE. Calculating the PIC involves 2 measurements: redundancy loss (*r*-loss) measuring parsimony and physical loss (*p*-loss) measuring precision. Lejarza and Baldea [22] proved that the coefficients of redundant terms are unstable in the moving horizon, thus resulting in a high coefficient of variation (*cv*). Therefore, the *r*-loss is calculated by the average *cv* of PDE coefficients for each combination (Fig. 1C, detailed in The moving horizon technique and calculation of *r*-loss in Materials and Methods). Considering that solving potential PDEs to obtain precision is nearly infeasible, an alternative way of utilizing PINN is adopted here. For the PINN, a correct physical constraint will improve performance (and vice versa), which indicates that if the potential PDE is inconsistent with the observed data, the outcome of PINN will deviate from the outcome of ANN with the incorrect physical constraint. For this reason, *p*-loss is measured by the deviation between the output of PINN and ANN (Fig. 1D, detailed in The PINN and calculation of *p*-loss in Materials and Methods). Finally, the PIC is calculated by the multiplication of *r*-loss and *p*-loss, and a smaller PIC indicates a better discovered PDE (Fig. 1E). The details of the PIC are provided in The PIC in Materials and Methods. After the ultimate structure of the PDE is determined by the PIC, the coefficients can be further optimized by the PINN to obtain better accuracy [19]. In essence, the PIC provides a measurement for each potential combination in order to select the most proper one. This means that the PIC does not involve a sophisticated optimization cycle to adjust physical constraints like existing PINN-based methods [18–20], and the PINN is merely employed to calculate *p*-loss where the physical constraint is fixed for each combination, which avoids the risk of nonconvergence.

### Discovery of canonical PDEs via the PIC

**Fig. 2.**
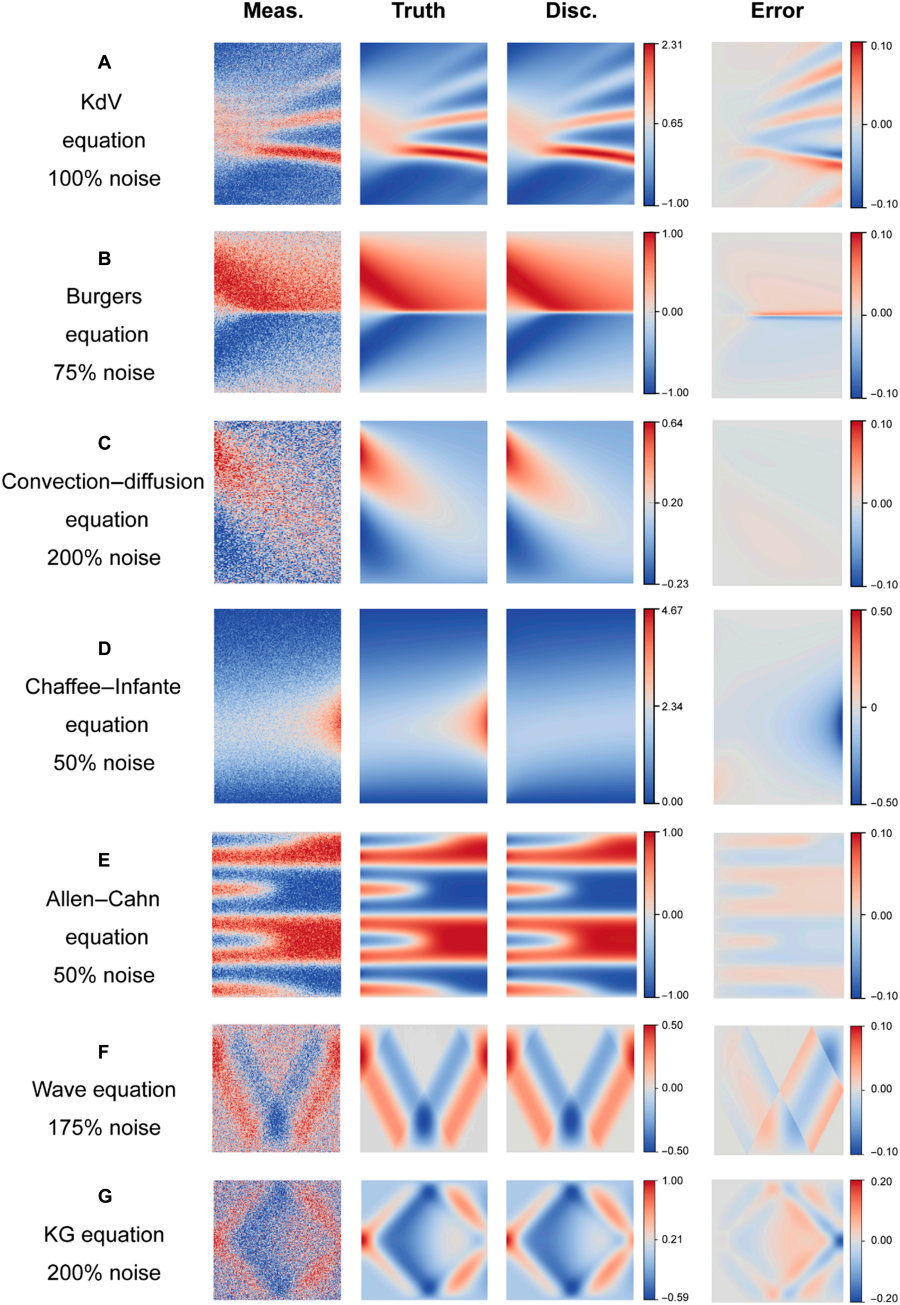
Discovery of canonical PDEs via the PIC with high levels of noise, including the KdV equation (A), the Burgers equation (B), the convection–diffusion equation (C), the Chaffee–Infante equation (D), the Allen–Cahn equation (E), the wave equation (F), and the KG equation (G). The noise level is the maximum noise where the correct PDE form can be discovered. Four columns from the left denote the noisy measurements (Meas.), the ground truth (Truth), the solution of the discovered PDE (Disc.), and the relative error (Error) defined as $\frac{\hat{u}-u}{\max\left(u\right)-\min\left(u\right)}$, where *u* is the ground truth and $\hat{u}$ is the solution of the discovered PDE. To facilitate comparison, the color bars of the first 3 columns are set to the same range for each case. For each picture, *x* is on the left and *t* is on the bottom.

In this section, we utilize the PIC to identify several canonical PDEs, including the Korteweg-De Vries (KdV) equation, the Burgers equation, the convection–diffusion equation, the Chaffee–Infante equation, the Allen–Cahn equation, the wave equation, and the Klein–Gordon (KG) equation. The descriptions and settings of these PDEs are provided in the Supplementary Materials (Sections S2.1 and S2.3). These PDEs originated from different physical fields, such as fluid mechanics and quantum mechanics. Here, the datasets are obtained from numerical solutions. In this work, the Gaussian noise is added as follows:

$$\tilde{u}=\varepsilon \cdot \mathrm{std}\left(u\right)\cdot N\left(0,1\right)+u,$$ (2)

where *u* are the clean data; $\tilde{u}$ are the noisy data; *N*(0,1) is the standard normal distribution; and *ε* is the noise level. In order to explore the robustness of the PIC to high noise, we increase the noise level by 25% until the correct PDE form fails to be discovered. We consider 2 types of activation functions, including the sin function [23] and the rational function [24], and select the better one when identifying each PDE. Additional details about these 2 activation functions are provided in the Supplementary Materials (Section S2.2). The results are displayed in Fig. 2 and the Supplementary Materials (Table S3). It is evident that the PIC method achieves outstanding performance when dealing with high levels of noise. Notably, in some cases, the PIC method is robust to extremely high noise (200% for the convection–diffusion equation and the KG equation and 175% for the wave equation). For most canonical PDEs, state-of-the-art (SOTA) robustness to data noise is obtained with high accuracy of discovered coefficients. Meanwhile, in most cases, the solution of the discovered PDE from PIC is close to the ground truth with low relative error, which further confirms the ability of the PIC to identify underlying physical processes from highly noisy data. Meanwhile, there are multiple types of noise, such as uniform noise [5], Gaussian noise [14], and random field noise. Mukhopadhyay et al. [25] has quantified the effect of different types of noise and revealed that the accuracy of the surrogate model is influenced by the type of noise. To evaluate and compare the performance and noise robustness of machine learning models and PIC under different noise types, we conducted experiments as outlined in the Supplementary Materials (Section S3.1). Our findings show that different noise types present diverse patterns, which influence the noise robustness of surrogate models.

**Fig. 3.**
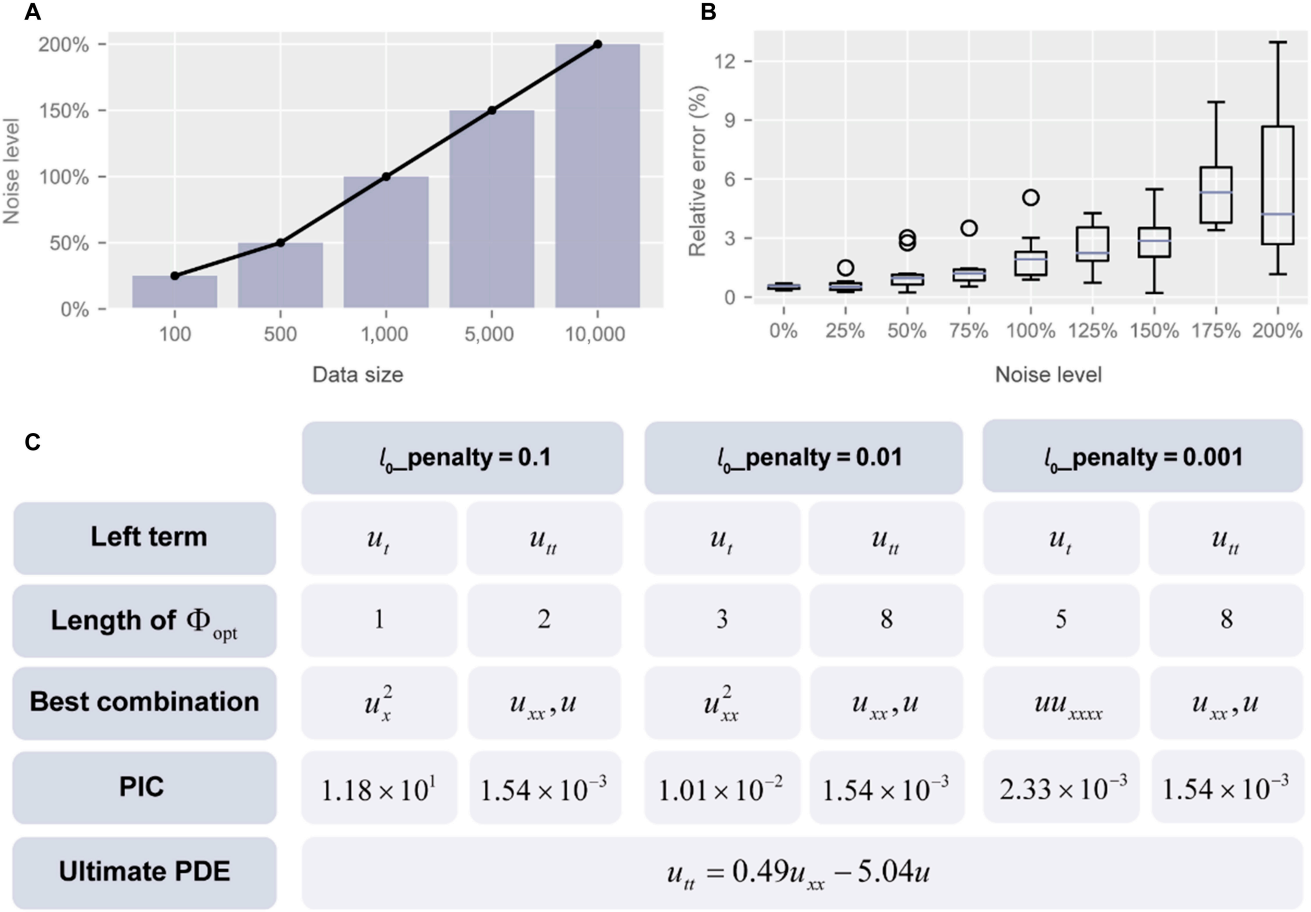
The robustness of the PIC to sparse data, high levels of noise, and hyperparameters of the penalty term. (A) The maximum noise where the PIC can discover the correct PDE form in different data sizes when identifying the convection–diffusion equation. (B) The boxplot of relative coefficient error for the ultimately discovered PDE from the data with different noise levels when identifying the KG equation with 10 different random seeds. The violet lines are the medians, and the black dots are the outliers. (C) The PIC and ultimate PDE when discovering the KG equation with 100% noise and different hyperparameters of the penalty term, i.e., *l_0_*_penalty, which controls the length of the preliminary potential terms Φ_opt_. Here, the LHS term *U_t_* is unknown, and the best combination discovered of the *u_t_* and *u_tt_* as the LHS term is compared to select the best one with a smaller PIC.

To further investigate the robustness of the PIC, the maximum noise where the PIC can discover the correct PDE form with different data sizes is provided in Fig. 3A. Here, the convection–diffusion equation is taken as an example. From the figure, the robustness to data noise increases with data size, and the performance is satisfactory with sparse data since it is still robust to 25% noise with merely 100 data points. Meanwhile, the relative coefficient error is utilized to measure the difference between the discovered PDE and the true PDE, which is defined as follows:

$$e_{\text{coef}}=\frac{1}{N_{\text{term}}}\sum_{i=1}^{N_{\text{term}}}\left|\frac{\xi_{i}-\xi_{i}^{\text{true}}}{\xi_{i}^{\text{true}}}\right|,$$ (3)

where *N_term_* is the number of PDE terms; and *ξ_i_* and $\xi_{i}^{\text{true}}$ are the coefficient of the discovered and the true PDE term, respectively. The relative coefficient error for different noise levels when identifying the KG equation is illustrated in Fig. 3B. Here, we conducted 10 independent experiments with different random seeds for each noise level to present a statistical result. It is evident that the relative error has an increasing trend with the noise level but remains relatively low with high levels of noise (only 4.21% median relative error for 200% noise).

Another experiment is conducted to examine the robustness of the hyperparameters of the penalty term. In most PDE discovery methods, the core hyperparameters that control the regularization or threshold markedly impact the discovered PDE since they decide the balance between parsimony and precision. The hyperparameters of the penalty term usually differ in different situations and have to be fine-tuned to obtain the correct PDE [26], which is unrealistic for practical applications. In the PIC, the most influential hyperparameter is the *l*_0_-penalty that determines the size of the preliminary library. In the generalized genetic algorithm, the fitness of discovered combinations is calculated by:

$$F=\mathrm{MSE}+l_{0}\_\text{penalty}\cdot N_{\text{term}},$$ (4)

where *F* denotes the fitness, which consists of *MSE* of the discovered combinations, and the *l_0_*_penalty term to penalize the number of terms. More details can be found in The generalized genetic algorithm optimization in Materials and Methods. We take the KG equation with 100% noise as an example, and the results with different magnitudes of *l*_0__penalty are provided in Fig. 3C. The LHS term *U_t_* is supposed to be unknown here, and the best combinations obtained with the hypothesis that the LHS term is *u_t_* or *u_tt_* are shown in the figure. It is seen that the size of the preliminary library increases when *l*_0__penalty decreases. However, the ultimately discovered PDE is stable for different magnitudes of *l*_0__penalty.

The high robustness and accuracy of the PIC are attributable to several aspects. Firstly, the neural network can smooth the data noise to some extent. The generalized genetic algorithm can provide a complete preliminary library since it has been proven to discover the redundant compensation terms to guarantee the inclusion of the correct dominant terms as far as possible in cases of high noise [19]. This issue is also confirmed in the Supplementary Materials (Section S3.2) by experiments. Secondly, the PIC takes both parsimony and precision into account, which can select the optimal PDE from numerous combinations from the preliminary library. Finally, the PIC essentially converts the NP-hard optimization problem into a finite-dimensional selection problem, which averts sophisticated adjustments of physical constraints, and thus improves robustness.

### Comparison with other methods and criteria

**Fig. 4.**
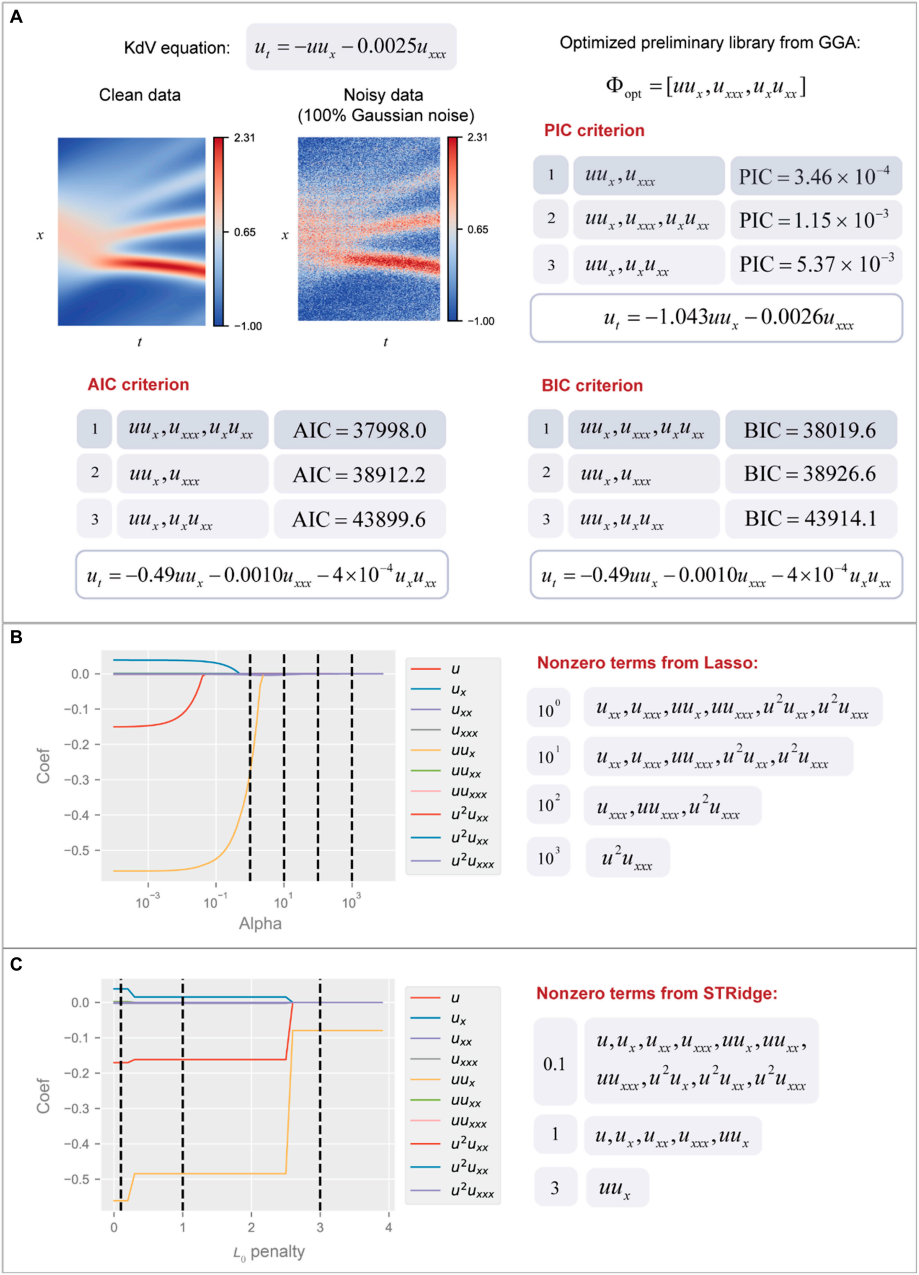
Comparison between commonly used criteria and methods. (A) The top 3 identified structures and ultimately discovered PDEs by the PIC (upper right), AIC (bottom left), and BIC (bottom right). (B) The nonzero terms discovered from the Lasso with different alpha, which controls the magnitude of *L*_1_ regulation. (C) The nonzero terms discovered from the STRidge with different *L*_0_ penalties. The above results are obtained when discovering the KdV equation with 100% noise. The black dashed line corresponds to the situation with different hyperparameters.

In this section, the PIC is compared with existing commonly used methods and criteria to demonstrate its superiority further. First, the PIC is compared with the Akaike information criterion (AIC) and the Bayesian information criterion (BIC) [27], which are often employed to measure the complexity and accuracy of the estimated model. We adopt them to identify optimal structures from the preliminary potential library to better demonstrate the difference between these information criteria. Here, the KdV equation with 100% noise is taken as an example, and the optimized preliminary library has 3 terms that make up 8 combinations. The top 3 discovered structures with these information criteria are shown in Fig. 4A. It can be seen that AIC and BIC have difficulty distinguishing the redundant terms, even if there are only 3 potential terms, when faced with high levels of noise. At the same time, the PIC can successfully identify the best structure since the PIC of the true structure is apparently smaller. Meanwhile, the coefficients obtained by the PIC are more accurate because of the PINN utilized in the PIC.

Afterwards, the PIC is compared with classic PDE discovery methods, including Lasso (utilizing *L*_1_ penalty) and STRidge (using *L*_2_ penalty and *L*_0_ penalty). Considering that both Lasso and STRidge require a complete candidate library, we utilize a basic candidate library with 10 terms:

$$\Phi =[u,u_{x},u_{\mathrm{xx}},u_{\mathrm{xxx}},{\mathrm{uu}}_{x},{\mathrm{uu}}_{\mathrm{xx}},{\mathrm{uu}}_{\mathrm{xxx}},u^{2}u_{x},u^{2}u_{\mathrm{xx}},u^{2}u_{\mathrm{xxx}}].$$ (5)

Here, the derivatives are also calculated from automatic differentiation. The KdV equation with 100% noise is taken as an example here. The discovered structures with different hyperparameters are demonstrated in Fig. 4B (Lasso) and Fig. 4C (STRidge). For the Lasso method, a larger alpha (*L*_1_ penalty) corresponds to a more parsimonious structure, but the true structure fails to be discovered with all magnitudes of alpha faced with highly noisy data. The STRidge method performs better since the correct dominant terms are contained in the discovered structure with different *L*_0_ penalties. However, the discovered structure has redundant terms with a small *L*_0_ penalty but lacks correct terms with a large *L*_0_ penalty. In contrast, the result of the PIC is both stable and accurate.

Meanwhile, the PIC is compared with prior works that also manage to discover these canonical PDEs under high levels of noise, including the PDE Robust Extraction Algorithm for Data method [18], the Robust Deep Learning-Genetic Algorithm method [19] and the Deep Learning-Genetic Algorithm method [5], and the results are provided in the Supplementary Materials (Table S4). Although PINN-based techniques [18,19] have achieved impressive performance, the PIC goes further with superior robustness and accuracy in most cases. It is worth noting that in the Burgers equation and the Allen–Cahn equation, the SOTA outcomes were previously obtained in a predetermined complete library with countable terms while the PIC searches the optimal PDE from unlimited potential combinations, which is more difficult yet closer to the reality.

### Extendibility and practical applications

In this section, the extendibility of the PIC to broader situations is investigated. The canonical PDEs examined in this work are all 1-dimensional cases, and the extension to higher dimensional conditions is straightforward and easy, details of which are provided in the Supplementary Materials (Section S1.1). Here, we adopt the PIC to discover the 2-dimensional (2D) Burgers equation with 200,000 discrete data points and different noise levels, the results of which are presented in Table. The true PDE form is written as follows:

$$u_{t}=-\left({\mathrm{uu}}_{x}+{\mathrm{uu}}_{y}\right)+0.01\left(u_{\mathrm{xx}}+u_{\mathrm{yy}}\right).$$ (6)

**Table.** The discovered PDE and corresponding PIC from data with different noise levels when identifying the 2D Burgers equation.

Noise level	Discovered equation	PIC
0%	*u_t_* = − 0.998(*uu_x_* + *uu_y_*) + 0.0093(*u_xx_* + *u_yy_*)	1.60 × 10^−5^
25%	*u_t_* = − 1.006(*uu_x_* + *uu_y_*) + 0.0095(*u_xx_* + *u_yy_*)	7.08 × 10^−6^
50%	*u_t_* = − 1.047(*uu_x_* + *uu_y_*) + 0.0093(*u_xx_* + *u_yy_*)	7.52 × 10^−6^
75%	*u_t_* = − 1.056(*uu_x_* + *uu_y_*) + 0.0092(*u_xx_* + *u_yy_*)	9.83 × 10^−6^
100%	*u_t_* = 0.133(*uu_xx_* + *uu_yy_*) − 0.040(*u_x_* + *u_y_*)	4.82 × 10^−5^

From the table, it is discovered that the PIC is robust up to 75% noise, and the discovered coefficient is accurate. The best results in existing works were achieved by Zhang and Liu [15], who utilize 515,100 grid data points, and their method is robust to 40% Gaussian noise. This indicates that the PIC can also discover high-dimensional PDEs with high accuracy and robustness. For a more challenging high-dimensional dynamic system, i.e., the 2D Navier–Stokes equation, the PIC has also demonstrated impressive performance. Specifically, in experiments with 400,000 (41.6%) discrete data points and up to 50% noise, the PIC method achieved a satisfactory level of accuracy, with an overall error of coefficients of approximately 5% (as outlined in Table S6). For more information and in-depth discussions of the experiment, please refer to the Supplementary Materials (Section S3.6).

We then investigate the extendibility of the PIC to discover parametric PDEs with variant coefficients, which is a challenge for most existing methods. The parametric convection–diffusion is taken as an example, the form of which is written as:

$$u_{t}=-\left(1+0.25\text{sin}\left(x\right)\right)u_{x}+u_{\mathrm{xx}}.$$ (7)

It is discovered that the PIC is robust to 50% noise (Table S5), which is a great improvement compared with the 25% noise obtained by Xu et al. [28] with the same condition. Furthermore, faced with 75% noise, although the coefficients’ form has a deviation, the correct structure is still discovered by the PIC. Additional details about the extension to high-dimensional PDEs and parametric PDEs can be found in the Supplementary Materials (Sections S1.1 and S2.6).

**Fig. 5.**
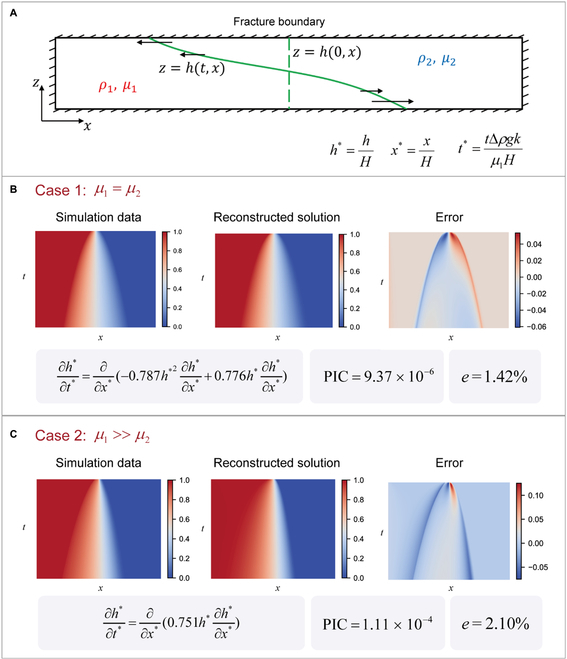
Practical applications of the PIC to discover unrevealed governing equations in proppant transport. (A) Simplified diagram of the physical process for proppant transport and dimensionless variable. The observation data are obtained from high-resolution microscopic simulation results. (B) The simulation data, the reconstructed solution from the discovered PDE, and the error in the case of *μ*_1_ = *μ*_2_. (C) The simulation data, the reconstructed solution from the discovered PDE, and the error in the case of *μ*_1_ ≫ *μ*_2_. *e* is the relative error.

Finally, the PIC is employed to discover unrevealed PDEs from a practical physical scene. In this work, we focus on a practical physical case of proppant transport, the process of which is illustrated in Fig. 5A. It describes the relative motion of 2 fluids with different densities and viscosities in a fracture or channel. The essence of the problem is similar to the viscous gravity current, which is an important natural phenomenon in geophysics. The asymptotic behavior of the developed current has been studied deeply in previous works [29], while the macroscale governing equation of the early-time behavior is difficult to be derived theoretically. Here, we will investigate the preasymptotic behavior at a macroscopic scale because of its importance in depicting proppant transport in fractures during the rapid process of hydraulic fracturing. As shown in Fig. 5A, the boundary between the 2 fluids is vertical under initial conditions, and the height of the boundary *h*(*x*) develops with time because of the influence of gravity. Here, we consider 2 situations, including *μ*_1_ = *μ*_2_ and *μ*_1_ ≫ *μ*_2_, where *μ*_1_ and *μ*_2_ are viscosities of fluid 1 and 2, respectively. The dataset *h*(*x*,*t*) is extracted from high-resolution microscopic simulation results and is standardized to obtain a general result. It is worth noting that the dataset is obtained from the numerical simulation at a microscopic scale [21], instead of solving known macroscale PDEs, because the theoretical governing equations at the macroscopic scale for this process have not been revealed previously. The details of the datasets are provided in the Supplementary Materials (Section S2.7). Here, the integration technique is adopted to facilitate discovery of the macroscale governing equations, which is detailed in the Supplementary Materials (Section S1.2). The ultimately discovered PDEs by the PIC are provided in Fig. 5B (*μ*_1_ = *μ*_2_) and Fig. 5C (*μ*_1_ ≫ *μ*_2_). The figure shows that the solution of the discovered PDE is consistent with the observation data with low error. Here, the relative error is defined as follows:

$$e={\left(\frac{\sum_{j=1}^{N_{t}}\sum_{i=1}^{N_{x}}{\left|h\left(x_{i},t_{j}\right)-h^{\prime }\left(x_{i},t_{j}\right)\right|}^{2}}{\sum_{j=1}^{N_{t}}\sum_{i=1}^{N_{x}}{\left|h\left(x_{i},t_{j}\right)\right|}^{2}}\right)}^{\frac{1}{2}}\times 100\%,$$ (8)

where *h*(*x_i_*, *t_j_*) are the observation data; *h*^′^(*x_i_*, *t_j_*) is the solution of the discovered PDE; and *N_x_* and *N_t_* are the number of *x* and *t*, respectively. The relative error for case 1 and case 2 is 1.42% and 2.10%, respectively, which indicates that the discovered PDE can fit the observed data very well. Furthermore, the discovered PDE is parsimonious and interpretable. For case 1, the discovered PDE can be rewritten as follows:

$$\frac{\partial h^{*}}{\partial t^{*}}=\frac{\partial }{\partial x^{*}}\left(0.776h^{*}\left(1-1.01h^{*}\right)\frac{\partial h^{*}}{\partial x^{*}}\right),$$ (9)

which can be seen as a parametric diffusion equation where the variant coefficients are related to the height h*, and the equation can be transferred into:

$$\frac{\partial h^{*}}{\partial t^{*}}=\frac{\partial }{\partial x^{*}}\left(\alpha \left(h^{*}\right)\frac{\partial h^{*}}{\partial x^{*}}\right),$$ (10)

where *α*(*h*^∗^) = 0.776*h*^∗^(1 − 1.01*h*^∗^). In essence, the variant coefficients characterize the diffusion trend at different heights. Meanwhile, it is surprising that the discovered PDE approximately satisfies the underlying symmetry *α*(*h*^∗^) = *α*(1 − *h*^∗^), which is consistent with the property of the physical process. Here, h* and 1− h* represent the height of fluid 1 and fluid 2, respectively, and if *μ*_1_ = *μ*_2_, they are equivalent and symmetry exists. Similarly, for case 2, the discovered PDE is also a parametric diffusion equation while the variant coefficient *α*(*h*^∗^) = 0.751*h*^∗^. The result can be explained physically because if *μ*_1_ ≫ *μ*_2_, the dynamic pressure of fluid 2 is much less than that of fluid 1, which means that fluid 1, with much larger viscosity, plays a leading role. Therefore, the variant coefficient is directly related to h* representing the height of fluid 1.

In general, the governing equations for the proppant transport process are discovered by the PIC, which were previously unrevealed. The discovered PDE can describe the physical process well and is consistent with the observation data from numerical simulation. Meanwhile, the discovered PDE is parsimonious and thus easy to be solved and explained, which advances the understanding and description of the preasymptotic behavior of the viscous gravity current. In addition, it also reveals underlying symmetries behind the physical process. The governing equations discovered by the PIC can facilitate the understanding and solution of the physical process because solving PDEs is much faster than high-resolution microscopic simulation. In prior work [21], the governing equations of the proppant transport process have also been discovered with small errors, which are written for the respective case 1 and case 2 as:

$$\frac{\partial h^{*}}{\partial t^{*}}=\frac{\partial }{\partial x^{*}}\left(0.894h^{*}\frac{\partial h^{*}}{\partial x^{*}}-0.881h^{*2}\frac{\partial h^{*}}{\partial x^{*}}+0.076{\left(\frac{\partial h^{*}}{\partial x^{*}}\right)}^{2}\right),$$ (11)

$$\frac{\partial h^{*}}{\partial t^{*}}=\frac{\partial }{\partial x^{*}}\left(1.006h^{*}\frac{\partial h^{*}}{\partial x^{*}}+0.215h^{*}{\left(\frac{\partial h^{*}}{\partial x^{*}}\right)}^{2}-0.571h^{*4}\frac{\partial h^{*}}{\partial x^{*}}\right).$$ (12)

It is obvious that complex redundant terms make the discovered PDE hard to be solved and explained, even more so regarding discovering symmetries, although the PDEs can describe the physical process, as well. By comparison, the PIC is able to provide more parsimonious results that give consideration to both accuracy and physical interpretability.

## Discussion

PDE discovery essentially constitutes a balance of precision and parsimony, which is controlled by regularization and threshold in previous literature. However, in terms of practical applications, problems will emerge in which it is difficult to determine the most proper PDE without the referenced PDE, since the results are usually different with different hyperparameters characterizing regularization. Therefore, in this work, a new criterion, the PIC, is proposed to determine the optimal PDE for both proof-of-concept cases and practical applications. SOTA outcomes are obtained for most canonical PDEs from different physical scenes, proving the PIC’s ability to deal with sparse and highly noisy data. The PIC method’s ability to tolerate high levels of noise can be attributed to 3 key factors. Firstly, the trained neural network smooths the noise to some extent. Secondly, the *r*-loss helps to distinguish redundant terms identified under high noise and provides some potential combinations. Lastly, the *p*-loss further reduces the impact of data noise and enables the method to more accurately distinguish the correct PDE from noisy data. In addition, the PIC method is employed in a specific physical case of proppant transport, which is essentially a viscous gravity current in the fracture. For this physical process, the unknown macroscale governing equation of the preasymptotic behavior is successfully discovered by the PIC from microscale simulation data without any other prior information. It is found that the discovered governing equation has good precision and interpretability and even satisfies potential symmetries, which facilitates the understanding and description of the preasymptotic behavior of the viscous gravity current. However, the computational cost of the PIC is currently higher than that of other methods, especially sparse regression-based methods, due to the need to train multiple neural networks. Moreover, the possible combinations increase with the size of the preliminary potential library with exponential growth, which is greatly time-intensive to traverse all possibilities when the size of the preliminary potential library is large. Nevertheless, the computation is affordable (usually taking 7 to 20 min in this work), and on-going efforts are attempting to optimize the PDE discovery process. In summary, the proposition of the PIC has achieved further satisfactory progress in practical applications of PDE discovery and will facilitate the discovery of unrevealed governing equations in broader physical scenes.

## Materials and Methods

### The generalized genetic algorithm optimization

In this work, a generalized genetic algorithm is utilized to provide the preliminary potential library, which originated from Xu and Zhang [19]. Here, we briefly introduce the process of the algorithm. As illustrated in Fig. 1B in the manuscript, the generalized genetic algorithm comprises several important steps, including translation, cross-over, mutation, fitness calculation, and evolution.

The PDE is digitized into genomes composed of inner terms and corresponding derivatives order in the translation step. For inner terms, the number refers to the corresponding derivative order, which is called basic genes. For example, 1 refers to *u_x_* for the right-hand side (RHS) term or *u_t_* for the LHS term, and 2 refers to *u_xx_* or *u_tt_*, similarly. The inner term is composed of the multiplication of basic genes. For example, (1,2) refers to the inner term *u_x_u_xx_*. Then, the term (or gene module) is constructed with the inner term and corresponding derivatives order. For example, [(1,2),1] refers to $\frac{\partial \left(u_{x}u_{\mathrm{xx}}\right)}{\partial x}$. Finally, several terms consist of the PDE by addition. For instance, {[(1,2),1], [(0,0),2]} refers to $\frac{\partial \left(u_{x}u_{\mathrm{xx}}\right)}{\partial x}+\frac{\partial^{2}\left(u^{2}\right)}{\partial x^{2}}$. Similarly, the LHS terms can be translated into genomes. With this digitization principle, each PDE corresponds to a specific genome, which paves the way for the subsequent process. The genetic algorithm is generalized because it can represent broader forms of terms, such as compound forms. For the initial generation, a number of genomes are randomly generated.

In the cross-over step, children are produced by swapping certain terms (or gene modules) in 2 parent genomes. In the mutation step, the children will mutate to generate new possibilities. There are 3 mutation ways, including the following: delete-module mutation, in which a randomly chosen module is deleted; add-module mutation, in which a randomly generated module is added; and basic gene mutation, in which a certain basic gene is replaced by a new randomly generated one. These 2 steps are crucial to the performance of the generalized genetic algorithm because unlimited combinations can be generated in these steps, which expands the search scope.

In the fitness calculation step, each genome’s fitness is calculated to measure the quality of genomes, which is defined as follows:

$$F=\mathrm{MSE}+l_{0}\_\text{penalty}\cdot N_{\text{term}},$$

with

$$\mathrm{MSE}=\frac{1}{N_{x}N_{t}}\sum_{j=1}^{N_{t}}\sum_{i=1}^{N_{x}}{\left|U_{L}\left(x_{i},t_{j}\right)-\vec{\xi }U_{R}\left(x_{i},t_{j}\right)\right|}^{2},$$ (13)

where *F* denotes the fitness, which consists of *MSE* part and *l_0_* penalty term; *MSE* is calculated according to Eq. 13, where *U_L_*(*x_i_*, *t_j_*) is the value of the LHS term with the size of 1 × 1; *U_R_*(*x_i_*, *t_j_*) is the value of RHS terms translated from the genome, which is an *N_term_*×1 vector; *N_term_* is the number of PDE terms; *N_x_* and *N_t_* are the number of *x* and *t* of the meta-data, respectively; and $\vec{\xi }$ with the size of 1×*N_term_* is the coefficient calculated by the least squares solution of $U_{L}-\vec{\xi }U_{R}=0$, which can be solved by the singular value decomposition method. Here, a smaller fitness indicates a better genome.

After the fitness has been calculated, half of the genomes with smaller fitness are reserved, and the others are replaced by new randomly generated genomes, which constitute the next generation. The evolution process will continue until the maximum iteration is achieved, and the optimal structure is the best genome with the smallest fitness in the last generation. It is worth noting that the compound form in the optimal structures will be decomposed and merged into the simplest form, which consists of the preliminary potential library.

Compared with other optimization methods, the generalized genetic algorithm possesses the advantage of broader representation and more robustness to data noise. It has been proven that the generalized genetic algorithm is able to discover the redundant compensation terms to guarantee the inclusion of the correct dominant terms [19], which is also demonstrated by an experiment provided in the Supplementary Materials (Section S3.2). The inclusion of the correct dominant terms is important for constructing a complete preliminary library from unlimited combinations, which paves the way for the PIC.

### The moving horizon technique and calculation of *r*-loss

The moving horizon is usually utilized for state and parameter estimation [30]. Lejarza and Baldea [22] first utilized this technique to facilitate model selection in ordinary differential equation discovery. In this work, the moving horizon technique is employed to calculate *r*-loss to measure the PDE’s parsimony. As illustrated in Fig. 1C in the manuscript, the smoothed meta-data are divided into *N_h_* overlapping horizons *T_i_*, which is defined as $\left[t_{\min}^{\prime }+i\Delta t,\frac{1}{2}\left(t_{\min}^{\prime }+t_{\max}^{\prime }\right)+i\Delta t\right]$, where $t_{\min}^{\prime }$ and $t_{\max}^{\prime }$ are the minimum and maximum of the time domain of the meta-data *t*^′^, respectively; *i*=1,2,...,*N_h_*; and Δ*t* is the length of horizons. The meta-data in horizons *T_i_* are generated from the neural network. For a given combination (i.e., PDE structure) Φ*_j_*, the optimal coefficients for each term in horizons *T_i_*, $\xi_{j}^{i}$, can be calculated by solving $U_{L}^{i,j}-\xi_{j}^{i}\cdot U_{R}^{i,j}=0,$ where $U_{L}^{i,j}$ and $U_{R}^{i,j}$ are the values of the LHS term and RHS terms for Φ*_j_* in *T_i_*, respectively. Therefore, for each term in Φ*_j_*, *N_h_* different coefficients are obtained, and the *cv* can be calculated as:

$${\mathrm{cv}}_{j,k}=\frac{\sigma_{j,k}}{\mu_{j,k}},$$ (14)

where *σ*_*j*, *k*_ and *μ*_*j*, *k*_ are the standard deviation and mean of the *N_h_* different coefficients for the *k^th^* term in Φ*_j_*, respectively. The *r*-loss of Φ*_j_* is calculated by the mean *cv* of all terms:

$$L_{r}\left(\Phi_{j}\right)=\frac{1}{N_{\text{term}}}\sum_{k=1}^{N_{\text{term}}}{\mathrm{cv}}_{j,k},$$ (15)

where *L_r_*(Φ*_j_*) is the *r*-loss for the combination Φ*_j_*; and *N_term_* is the number of terms. With the moving horizon, the *r*-loss can measure parsimony well because the redundant terms function as the compensation for the error caused by noise, which is different in different horizons, leading to a high *cv*. In contrast, the correct dominant terms are stable with smaller *cv*. Additional details and discussions are provided in the Supplementary Materials (Section S3.3).

### The PINN and calculation of *p*-loss

The PINN was first proposed by Raissi [17] to construct surrogate models and improve prediction performance. Compared with common neural networks, the PINN adds physical constraints to the loss function of the neural network to make the prediction fit the prior physical knowledge well. As illustrated in Fig. 1D in the manuscript, in this work, the PINN is utilized to measure the precision of the discovered PDE without solving the PDE numerically. The construction of the PINN is the same as ANN, which consists of 1 input layer, several hidden layers, and 1 output layer, while the loss of physical constraint is added to the loss function:

$$L_{\text{PINN}}\left(\theta \right)=\lambda_{\text{Data}}{\mathrm{MSE}}_{\text{Data}}+\lambda_{\mathrm{PDE}}{\mathrm{MSE}}_{\mathrm{PDE}},$$

$${\mathrm{MSE}}_{\text{Data}}=\frac{1}{N_{x}N_{t}}\sum_{i=1}^{N_{x}}\sum_{j=1}^{N_{t}}{\left(u\left(x_{i},t_{j}\right)-\text{PINN}\left(x_{i},t_{j};\theta \right)\right)}^{2},$$ (16)

$${\mathrm{MSE}}_{\mathrm{PDE}}=\frac{1}{N_{x}^{\prime }N_{t}^{\prime }}\sum_{i=1}^{N_{x}^{\prime }}\sum_{j=1}^{N_{t}^{\prime }}{\left(U_{L}^{\prime }\left(x_{i}^{\prime },t_{j}^{\prime }\right)-\xi^{\prime }\cdot U_{R}^{\prime }\left(x_{i}^{\prime },t_{j}^{\prime }\right)\right)}^{2}.$$

where *L_PINN_*(*θ*) is the loss function of PINN, which consists of data loss (*MSE_Data_*) and PDE loss (*MSE_PDE_*); and *θ* is the parameters of PINN, including the weights and bias. The data loss is the mean squared error (*MSE*) of the difference between observed data and predicted data by PINN. The PDE loss is the *MSE* of the difference between the LHS term $U_{L}^{\prime }$ and the RHS term $\xi^{\prime }\cdot U_{R}^{\prime }$ of the PDE. Here, the PDE loss is calculated on the meta-data $\left(x_{i}^{\prime },t_{j}^{\prime }\right)$ generated from the neural network. It is worth noting that the coefficients of the PDE *ξ*^′^ are calculated by solving $U_{L}^{\prime }-\xi^{\prime }\cdot U_{R}^{\prime }=0$ in each training epoch. In this work, the PINN is trained on the basis of the pretrained ANN in Fig. 1A in the manuscript, and the input is the observation data (*x_i_*, *t_j_*), as well. The output of PINN on the meta-data is *u*^PINN^, while the output of the pretrained ANN on the meta-data is *u*^ANN^. To calculate *p*-loss, we standardize the output by:

$${\hat{u}}^{\text{PINN}}=\frac{1}{u_{\max}-u_{\min}}\left(u^{\text{PINN}}-u_{\min}\right),$$ (17)

$${\hat{u}}^{\mathrm{ANN}}=\frac{1}{u_{\max}-u_{\min}}\left(u^{\mathrm{ANN}}-u_{\min}\right),$$

where *u_max_* and *u_min_* are the maximum and minimum of the observation data, respectively. Then, the *p*-loss is calculated by the root mean squared error between the standardized output of PINN and ANN, which is denoted as follows:

$$L_{p}\left(\Phi_{j}\right)={\left(\frac{1}{N_{x^{\prime }}N_{t^{\prime }}}\sum_{m=1}^{N_{x^{\prime }}}\sum_{n=1}^{N_{t^{\prime }}}{\left({\hat{u}}_{m,n}^{\text{PINN}}-{\hat{u}}_{m,n}^{\mathrm{ANN}}\right)}^{2}\right)}^{\frac{1}{2}}.$$ (18)

In this work, we use the PINN to measure the precision of the discovered PDE. It is based on the fact that the physical constraint has an influential impact on the prediction. If the physical constraint is parallel with the data, the prediction will be improved. However, if the physical constraint and the data are inconsistent, the training will be affected greatly, and the result will derive greatly from the output of ANN. Since ANN’s output is relatively accurate, the *p*-loss will be very small if the PDE can describe the data. Therefore, the *p*-loss can measure precision efficiently without solving PDEs.

### The PIC

As illustrated in Fig. 1E in the manuscript, the PIC is defined by the multiplication of the calculated *p*-loss and *r*-loss, which is written as:

$$\mathrm{PIC}=L_{r}\left(\Phi_{j}\right)\cdot L_{p}\left(\Phi_{j}\right).$$ (19)

Here, a small PIC means that the PDE has both better parsimony and precision. The whole PIC algorithm is detailed below. In this work, an ANN is pretrained to construct a surrogate model to generate smoothed meta-data and calculate derivatives. Then, the generalized genetic algorithm optimization is employed to obtain an optimal structure that forms the preliminary potential library Φ_opt_. For the preliminary potential library with countable terms, the *r*-loss of all possible combinations is calculated. Here, if the preliminary library has *N_opt_* terms, the total number of combinations is 2*^N_opt_^*. The potential library is unlimited in common practice, which poses an NP-hard problem. This is why we utilize the generalized genetic algorithm in this work to obtain a countable preliminary library. It is worth noting that the size of the preliminary library is usually smaller than 10, and the computational cost is reasonable because the calculation process of *r*-loss is quick for each combination. After the *r*-loss has been calculated, we select the top *N_b_* combinations with smaller *r*-loss and further calculate *p*-loss. Considering that the training of PINN is time-consuming, it is infeasible to calculate *p*-loss of all combinations. Afterwards, the PIC of the *N_b_* combinations is calculated, and the ultimately discovered PDE is the combination with the smallest PIC. Finally, the coefficient of the discovered PDE is further optimized by the PINN.

## Data Availability

All datasets utilized in this work are available on GitHub. The URL is https://github.com/woshixuhao/PIC_code/tree/main/data. The source code is available on GitHub. The URL is https://github.com/woshixuhao/PIC_code.

## Supplementary Materials

Supplementary 1 Sections S1 to S3. Tables S1 to S6. Figs. S1 to S5. References [31–34]
