# Supplementary material for: Discovery of Partial Differential Equations from Highly Noisy and Sparse Data with Physics-Informed Information Criterion
Source: Research (Wash D C). 2023 May 19;6:0147. doi: 10.34133/research.0147 (PMC10198462; doi:10.34133/research.0147)
Supplement: Supplementary 1 — Sections S1 to S3. Tables S1 to S6. Figs. S1 to S5. References [31–34] [file research.0147.f1.docx]

**Supplementary Material for**

Discovery of partial differential equations from highly noisy and sparse data with physics-informed information criterion

Hao Xu^a^, Junsheng Zeng^b^, and Dongxiao Zhang^c,d,e,*^

^a^ *BIC-ESAT, ERE, and SKLTCS, College of Engineering, Peking University, Beijing 100871, P. R. China*

^b^ *Institute of Applied Physics and Computational Mathematics, Beijing 100088, P. R. China*

^c^ *Eastern Institute for Advanced Study, Eastern Institute of Technology, Ningbo315200, Zhejiang, P. R. China*

^d^ *National Center for Applied Mathematics Shenzhen (NCAMS), Southern University of Science and Technology, Shenzhen 518055, Guangdong, P. R. China*

^e^ *Department of Mathematics and Theories, Peng Cheng Laboratory, Shenzhen 518000, Guangdong, P. R. China*

^*^ Corresponding author

Email address: [390260267@pku.edu.cn](file:///C:\Users\xh251314\Documents\WeChat%20Files\wxid_zctlb8310gyu22\FileStorage\File\2022-12\390260267@pku.edu.cn) (H. Xu); [zengjs1993@163.com](mailto:zengjs1993@163.com) (J. Zeng); [zhangdx@sustech.edu.cn](file:///C:\Users\xh251314\Documents\WeChat%20Files\wxid_zctlb8310gyu22\FileStorage\File\2022-12\zhangdx@sustech.edu.cn) (D. Zhang).

**1. Details for methods**

**1.1 Extensibility to high-dimensional PDEs and parametric PDEs**

The PIC proposed in this work can be extended to different situations with only a slight adjustment. For high-dimensional PDEs, the derivatives of all dimensions are added to the basic genes of the generalized genetic algorithm, which leads to a preliminary library with high-dimensional terms. Here, we take the extension to 2D cases as an example, and the problem is converted into the following:

$U_{t}=\Omega(u,u_{x},uu_{x},u_{xx},u_{y},uu_{y},u_{yy}...)\cdot\vec{\xi},$ (S.1)

where interactive terms, such as *u_x_* and *u_y_*, are not considered. For high-dimensional problems, considering that the search space is too large to be optimized, symmetry is always employed to facilitate the optimization and covert the problem into:

$U_{t}=\Omega(u,\nabla u,(u\cdot\nabla)u,\nabla^{2}u,...)\cdot\vec{\xi},$ (S.2)

where the symmetrical terms are considered. For the generalized genetic algorithm, the basic genes are composed of the basic symmetrical terms, such as $u,\nabla u,\nabla^{2}u,$ etc. To maintain symmetry, we do not consider the compound form here. After these treatments, the PIC can be adapted to high-dimensional cases in a manner similar to the 1D case.

For parametric PDEs, the PIC can also assist to improve robustness based on the existing method. The core issue of identifying parametric PDEs is determining the PDE structure. In previous work [28], the optimal structure is identified by local windows and the voting principle, which may be ineffective when the noise level is high. Here, the PIC is adapted to improve the discovery of parametric PDEs. In each local window, a preliminary library can be obtained from the generalized genetic algorithm, which can be combined into a summarized library for the global domain. Then, the *r*-loss of all possible combinations of the summarized library is calculated, and the *p*-loss of several top combinations is calculated. The PDE loss is slightly different since the coefficient is variant. Finally, the ultimate structure is discovered, and the variant coefficients are optimized in PINN. Furthermore, the curves of the variant coefficients can be fitted by the DLGA-PDE (coefficient) proposed by Xu et al. [28].

**1.2 Discovery of the governing equation of proppant transport by the PIC**

In this work, the PIC is employed to discover the unrevealed governing equation of proppant transport. Here, we first briefly introduce the background of the physical scene. The proppant transport problem originated from the hydraulic fracturing process where the fluid is injected into a crack, and the fluid pressure is the driving force for the fracture opening and propagation [31]. The proppant is used to prevent the fracture from closing once the well is depressurized, and the transport of the proppant is affected by the original liquid in the crack. In this work, we consider a simplified situation to better reveal the process’s essence. The dataset is generated from 2D high-resolution microscopic simulations with closed boundaries in a homogeneous porous medium or rectangle vertical fracture. The permeability and porosity are considered constant. In case 1 where $\mu_{1}=\mu_{2}$, the dataset is grid data of 500 spatial observation points in the domain $x\in[0,500)$ and 500 temporal observation points in the domain $t\in[0,100)$, and thus the data size is 250,000. In case 2 where $\mu_{1}\gg\mu_{2}$, the temporal domain changes to be $t\in[0,1000)$, while other conditions remain the same. It is worth noting that the preasymptotic behavior is investigated here. As illustrated in Fig. 5a in the manuscript, the dimensionless variable is employed to discover a general PDE. We randomly select 200,000 data to train the neural network.

In this actual complex problem, some techniques are adopted to facilitate the discovery of governing equations. Firstly, considering that the system is conservative, the underlying governing equations can be transformed into the conservative form:

$\frac{\partial h^{*}}{\partial t^{*}}+\frac{\partial F^{*}(x^{*},t{}^{*})}{\partial x^{*}}=0,$ (S.3)

where *F^*^* is the dimensionless flux related to *h^*^* and its derivatives. Therefore, the flux *F* can be calculated by integration techniques as follows:

$F^{*}(x^{*},\text{ }t{}^{*})=\int_{x_{0}}^{x^{*}} -\frac{\partial h^{*}}{\partial t^{*}}dx^{*}.$ (S.4)

Benefiting from the neural network, the *F^*^* on the meta-data can be calculated easily. Therefore, the problem is converted to discover the PDE form of the dimensionless flux *F^*^*, which is accomplished by the PIC in this work.

**2. Experimental settings**

For the reproducibility of this work, we provide detailed experimental settings, including information about the methods, parameters and data, in this section.

**2.1 The canonical PDEs in this work**

In this work, seven canonical PDEs from different physical fields are investigated to examine the robustness and accuracy of the proposed PIC for proof-of-concept. Here, we will briefly introduce these PDEs.

1. The Korteweg-De Vries (KdV) equation, which is utilized to describe the evolution of 1D shallow-water waves, is written as:

$$u_{t}=-uu_{x}-0.0025u_{xxx}.$$

The dataset is grid data of 512 spatial observation points in the domain $x\in[-1,1)$ and 201 temporal observation points in the domain $t\in[0,1]$, and thus the data size is 102,912.

1. The Allen-Chan equation, which is a nonlinear reaction-diffusion equation that describes the phase separation of multi-component metal alloys [32], is written as:

$$u_{t}=0.003u_{xx}+u-u^{3}.$$

The dataset is grid data of 256 spatial observation points in the domain $x\in(-1,1)$ and 201 temporal observation points in the domain $t\in(0,10]$, and thus the data size is 51,456.

1. The wave equation, which is a commonly-used PDE to describe the vibration and fluctuation phenomenon, is written as:

$$u_{tt}=u_{xx}.$$

The dataset is grid data of 161 spatial observation points in the domain $x\in[0,\pi]$ and 321 temporal observation points in the domain $t\in[0,2\pi]$, and thus the data size is 51,681.

1. The convection-diffusion equation, which can be employed to describe the transport of substance in a fluid (e.g., contaminant transport), is written as:

$$u_{t}=-u_{x}+0.25u_{xx}.$$

The dataset is grid data of 256 spatial observation points in the domain $x\in[0,2]$ and 100 temporal observation points in the domain $t\in[0,1]$, and thus the data size is 25,600.

1. The Burgers equation, which has a wide application in many fields, including fluid mechanics, nonlinear acoustics, gas dynamics and traffic flow [33], is written as:

$$u_{t}=-uu_{x}+0.1u_{xx}.$$

The dataset is grid data of 256 spatial observation points in the domain $x\in(-8,8)$ and 201 temporal observation points in the domain $t\in(0,10]$, and thus the data size is 51,456.

1. The Klein-Gordon (KG) equation, which was first proposed by Oskar Klein and Walter Gordon in 1926 to describe the behavior of electrons in relativistic settings, is written as:

$u_{tt}=0.5u_{xx}-5u$.

The dataset is grid data of 201 spatial observation points in the domain $x\in[-1,1]$ and 201 temporal observation points in the domain $t\in[0,3]$, and thus the data size is 40,401.

1. The Chaffee-Infante equation, which is widely used in numerous fields, such as environmental science, fluid dynamics, high-energy physics and electronic science [5], is written as:

$u_{t}=u_{xx}-u+u^{3}$.

The dataset is grid data of 301 spatial observation points in the domain $x\in[0,3]$ and 200 temporal observation points in the domain $t\in(0,0.5)$, and thus the data size is 60,200.

**2.2 The settings of the neural network and the physics-informed neural network (PINN)**

This work uses a fully connected artificial neural network (ANN) to construct a surrogate model, generate meta-data, and calculate derivatives via automatic differentiation. For the ANN, the number of hidden layers is 5, with 50 neurons in each hidden layer, the number of input neurons is 2, and the number of output neurons is 1. The input is the spatial-temporal location (*x*,*t*), and the target is the observation *u*. The activation function is the Sin function [17,23], *f*(*x*)=sin(*x*), or the Rational function [24], which is written as:

$f(x)=\frac{P(x)}{Q(x)}=\frac{\sum_{i=1}^{r_{P}} a_{i}x^{i}}{\sum_{j=1}^{r_{Q}} b_{j}x^{j}},$ (S.5)

where *P*(*x*) and *Q*(*x*) are polynomials of *x* with the order of *r_P_* and *r_Q_*, respectively. In this work, *r_P_* and *r_Q_* are 3 and 2, respectively. The Rational activation function is proven to be more suitable for some cases with high noise [18]. Therefore, in this work, we compare the result of both activation functions and select the best one for each case. The optimizer is chosen to be Adam with a learning rate of 0.001. The maximum training epoch is 30,000, and the early stop technique is adopted to prevent overfitting based on the training loss and validating loss. The construction of the physics-informed neural network (PINN) is the same as the ANN, with different loss functions mentioned in the Methods section of the main text. For calculating the physical loss (*p*-loss), the PINN is trained for 300 epochs based on the pre-trained neural network for each combination. The $\lambda_{Data}$ and $\lambda_{PDE}$ are 1 and 0.01, respectively, which are decided empirically. After the ultimate PDE is determined, the coefficients are optimized by PINN. Here, the training epoch of PINN is 3,000 for the KG equation and the wave equation, and 1,000 for the others.

**2.3 The settings of the meta-data**

Benefitting from the neural network, we can construct a surrogate model from a few sparse, noisy data to generate meta-data on grids, which plays a vital role in the generalized genetic algorithm and calculation of *r*-loss and *p*-loss. Here, we provide information on the meta-data for the PDEs utilized in this work. In all cases, the meta-data for the generalized genetic algorithm and *r*-loss are grid data of 100 spatial observation points and 100 temporal observation points, and thus the data size is 10,000. For the PINN, the test data for calculating *p*-loss are grid data of 100×100 for the Rational activation function and 200×200 for the Sin activation function. The domain of the meta-data in each PDE is detailed in Table S1.

**Table S1.** The spatial and temporal domains of the meta-data for the canonical PDEs utilized in this work. $x'$ and $t'$ are the generated meta-data in spatial and temporal domain, respectively.

| **Equation name** | **Equation form** | **Spatial domain** | **Temporal domain** |
| --- | --- | --- | --- |
| KdV equation | $u_{t}=-uu_{x}-0.0025u_{xxx}$ | $x'\in[-0.8,0.8]$ | $t'\in[0.1,0.9]$ |
| Burgers equation | $u_{t}=-uu_{x}+0.1u_{xx}$ | $x'\in[-7,7]$ | $t'\in[1,9]$ |
| Convection-diffusion equation | $u_{t}=-u_{x}+0.25u_{xx}$ | $x'\in[0,2]$ | $t'\in[0,1]$ |
| Chaffee-Infante equation | $u_{t}=u_{xx}-u+u^{3}$ | $x'\in[0.5,2.5]$ | $t'\in[0.15,0.45]$ |
| Allen-Cahn equation | $u_{t}=0.003u_{xx}+u-u^{3}$ | $x'\in[-0.8,0.8]$ | $t'\in[1,9]$ |
| Wave equation | $u_{tt}=u_{xx}$ | $x'\in[0.1,3]$ | $t'\in[0.2,6]$ |
| KG equation | $u_{tt}=0.5u_{xx}-5u$ | $x'\in[-0.8,0.8]$ | $t'\in[0.3,2.7]$ |

It can be seen that the domain of meta-data is slightly narrower than that of the observation data, which is because the derivatives near the boundary are relatively inaccurate. Therefore, we avoided the original boundary when generating meta-data.

**2.4 The settings of the moving horizon**

When obtaining the *r*-loss of the combinations, the smoothed meta-data are divided into *N_h_* overlapping horizons *T_i_*, which is defined as , where $t'_{\text{min}}$ and $t'_{\text{max}}$ are the minimum and maximum of the time domain of the meta-data $t'$, respectively; *i*=1,2,...,*N_h_*; and $\Delta t$ is the length of horizons. In this work, $\Delta t$ is set to be:

(S.6)

In this work, *N_h_* is 10.

**2.5 The settings for the generalized genetic algorithm**

In this section, we provide the settings for the generalized genetic algorithm. In this work, the population size of genomes is 400, and the number of maximum generations is 200. The rate of cross-over is 1.0, the rate of add mutation and the basic gene mutation is 0.4, and the rate of delete module mutation is 0.5. The basic genes are *u*, *u_x_*, *u_xx_* and *u_xxx_*, and the derivative order is considered up to 3. This means that the maximum derivative order for terms can be up to 6 (e.g., $\frac{\partial^{3}u_{xxx}}{\partial x^{3}}$), which is sufficient for most situations. The *l_0_* penalty in the fitness is 0.1 for the KdV equation, the Chaffee-Infante equation, and the KG equation, 0.01 for the wave equation and the convection-diffusion equation, and 0.0005 for the Burgers equation and the Allen-Cahn equation. The hyper-parameter *l_0_* penalty is selected according to trial and error, and the selection is not restricted (proven in the main text). A proper *l_0_* penalty leads to a preliminary library with a suitable number of terms (usually fewer than 10 terms), which will conserve the calculation cost when calculating *r*-loss.

**2.6 The setting of the experiment examining the extendibility of the PIC**

In this work, the 2D Burgers equation and the parametric convection-diffusion equation are employed to show the extendibility of the proposed PIC. Here, information about these two PDEs is provided. The visualization of both datasets is provided in Fig. S1.

For the 2D Burgers equation, the dataset is the same as that utilized in Zhang and Liu [15], where the initial condition is $u(x,y,0)=0.1\text{sech}(20x^{2}+25y^{2})$, and the boundary condition is periodic. The dataset is the grid data of 101 spatial observation points in the domain$x\in[-1,1]$, 51 spatial observation points in the domain$y\in[-1,1]$, and 100 temporal observation points in the domain $t\in[0,2)$. The data size is 515,100. The meta-data are the grid data of 20 spatial observation points in the domain$x\in[-0.8,0.8]$, 20 spatial observation points in the domain$y\in[-0.8,0.8]$, and 20 temporal observation points in the domain $t\in[0,2]$. The meta-data size is 8,000. In this work, 200,000 discrete data are randomly selected from the dataset to construct the training data.

For the parametric convection-diffusion equation, the form is written as:

$u_{t}=-(1+0.25\sin(x))u_{x}+u_{xx}.$ (S.7)

The dataset is the same as that in Xu et al. [28], which has 250 temporal observation steps in $t\in[0,5)$and 201 spatial observation steps in $x\in[0,8]$, and thus the data size is 50,250. The meta-data are grid data of 100 spatial observation points in the domain$x\in[0.5,7.5]$ and 100 temporal observation points in the domain $t\in[0.2,4.8]$. In this work, 30,000 discrete data are randomly selected from the dataset to construct the training data. The discovery of parametric PDEs by the PIC is based on previous research [28], which utilizes stepwise methods to identify the PDE structure and then employs the PINN to calculate the variant coefficients. Finally, the expression of coefficients is discovered by the DLGA-PDE (coefficients) [28]. In this work, a preliminary library can be obtained from the generalized genetic algorithm in each local window, which can be combined into a summarized library for the global process. Then, the *r*-loss of all possible combinations of the summarized library is calculated, and the *p*-loss of several top combinations is calculated. The PDE loss is calculated by:

$$L_{PINN}(\theta)=\lambda_{Data}MSE_{Data}+\lambda_{PDE}MSE_{PDE},$$

$MSE_{Data}=\frac{1}{N_{x}N_{t}}\sum_{i=1}^{N_{x}} \sum_{j=1}^{N_{t}} (u(x_{i},t_{j})-PINN(x_{i},t_{j};\theta)),\text{ }$ (S.8)

The difference is that the $\xi'_{i}$ in the *MSE_PDE_* is a variable, and the value on each *x* is calculated by solving the optimal solution of by least squared regression.

**
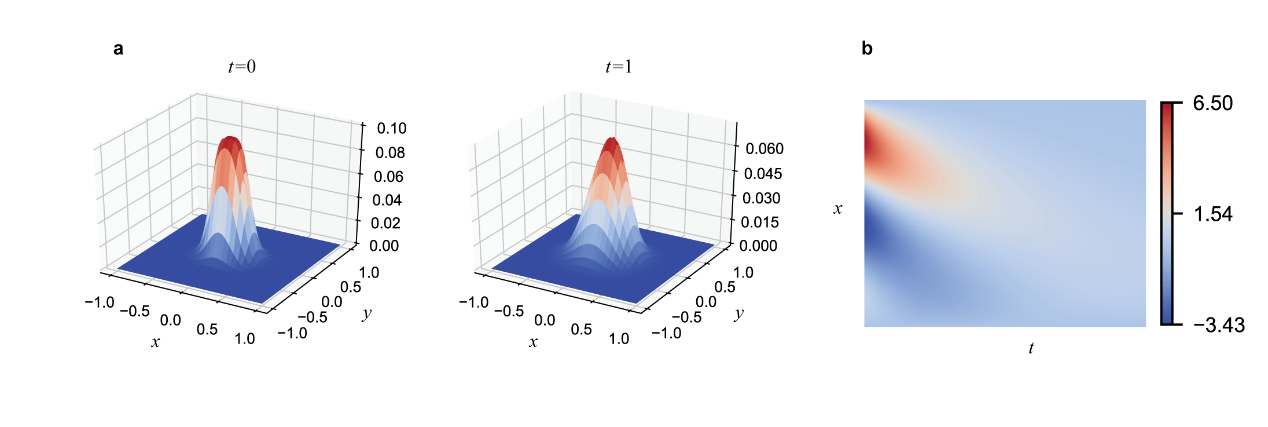
 Fig. S1. Visualization of the dataset for the 2D Burgers equation (a) and the** **parametric convection-diffusion equation (b)**. **a,** The 2D Burgers equation. The 2D surface of *u*(*x,y*) when *t*=0 and *t*=1 is provided. **b,** The heatmap of *u*(*x,t*) for the parametric convection-diffusion equation.

**2.7 The dataset for proppant transport**

In this work, the PIC is employed in a specific physical case of proppant transport, which is essentially a viscous gravity current in the fracture. The dataset is generated from microscale simulation in two situations, including $\mu_{1}=\mu_{2}$ and $\mu_{1}\gg\mu_{2}$, where $\mu_{1}$ and $\mu_{2}$ are viscosities of fluid 1 and 2, respectively. The visualization of the simulation data is depicted in Fig. S2.


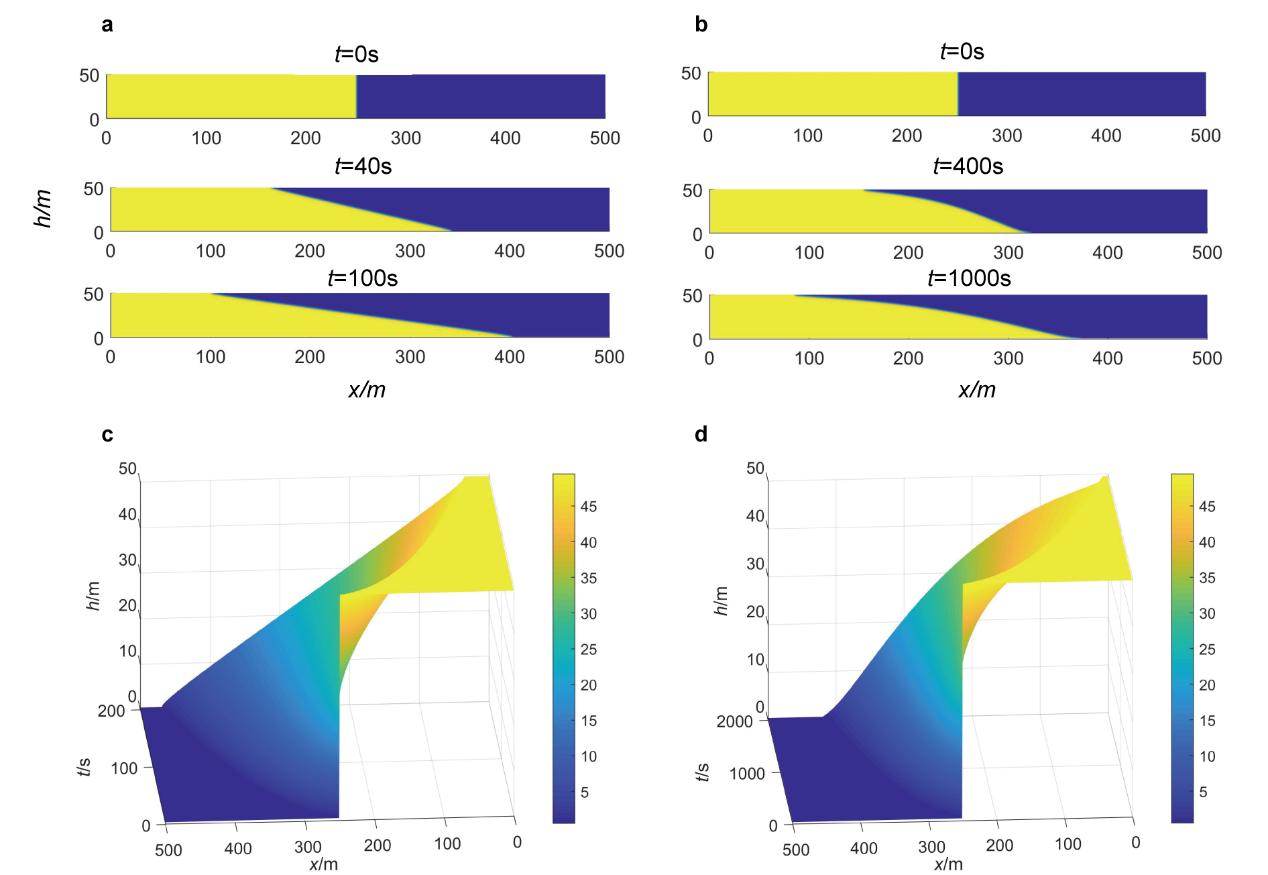


**Fig. S2. Visualization of the datasets for proppant transport.** **a** and **b** are the evolution of interface for $\mu_{1}=\mu_{2}$ and $\mu_{1}\gg\mu_{2}$ cases, respectively. **c** and **d** are the 3D heatmaps of the data for $\mu_{1}=\mu_{2}$ and $\mu_{1}\gg\mu_{2}$ cases, respectively.

**3. Supplementary experiments**

**3.1 The influence of data noise**

In this work, the robustness of the proposed PIC to data noise is examined, and satisfactory results are obtained, in which the PIC is robust to high levels of Gaussian noise with sparse, discrete data. In this section, we investigate the performance of the PIC with different types of data, including Gaussian noise, uniform noise, and random field noise. Gaussian noise is a commonly used noise type in previous works [15], while uniform noise is another noise type in PDE discovery works [34]. Both Gaussian and uniform noise are white noise. In contrast, random field noise is a non-white noise rarely seen in previous investigations, which is more challenging for PDE discovery and is more realistic. The KdV equation is taken as an example here, and the maximum noise to which the PIC can be robust is illustrated in Fig. S3.

**
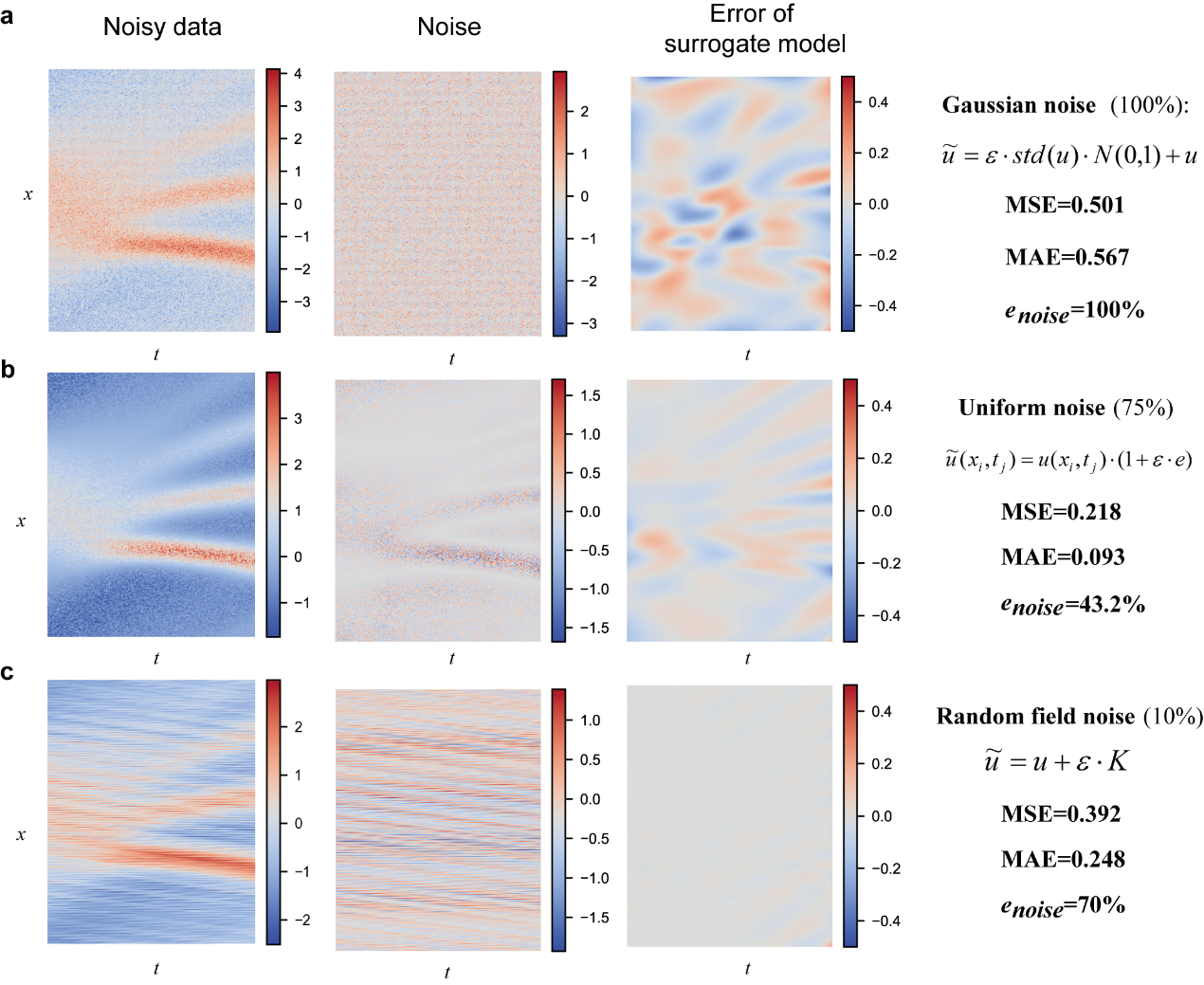
Fig. S3. Comparison between different types of noise.** **a,** Gaussian noise. **b,** Uniform noise. **c,** Random field noise. The KdV equation is taken as an example here, and the noise level is the maximum noise that the PIC can handle. The left is the noisy data, the middle is the visualization of noise, and the right is the error of the surrogate model. *K* is a Gaussian random field with a correlation length of 32.

In order to better compare these types of noise, the MSE, MAE, and relative error of the noise *e_noise_* are calculated as follows:

(S.9)

where *u* are the clean data; $\tilde{u}$are the noisy data; and *N_x_* and *N_t_* are the number of *x* and *t* of the data, respectively. From the figure, it is found that the PIC can handle these three types of noise well, which confirms the robustness of the PIC to different types of noise. Meanwhile, it can be seen that the MSE, MAE, and *e_noise_* of the Gaussian noise are the largest among these three types of noise, which means that the PIC is more suitable to handle Gaussian noise. It is interesting to find that the uniform noise is harder to deal with than the Gaussian noise. The reason for this may be that the uniform noise greatly influences the major part of the physical process. In contrast, the Gaussian process affects the entire space-time domain, which is easier to smooth by the neural network. Meanwhile, the error of the surrogate model is illustrated in Fig. S3, and it is evident that different noise types present diversified patterns, which influence the noise robustness of the surrogate model.

**3.2 The effectiveness of the generalized genetic algorithm**

The generalized genetic algorithm is utilized in this work to obtain a preliminary potential library. It is crucial for the preliminary library to contain all correct terms to be a complete library. Therefore, the ability of the generalized genetic algorithm to guarantee the correct terms to be included should be examined. In previous research [19], it has been proven that high-order redundant compensation terms can be discovered by the generalized genetic algorithm, which can compensate for the error from noise and guarantee the inclusion of the correct dominant terms. Here, we also conduct an experiment to confirm this issue. Here, the KdV equation with 100% Gaussian noise is taken as an example, and the results are provided in Table S2. The table shows that the coefficients calculated by direct least squares regression on the true terms derived primarily from the true coefficients. In contrast, although the generalized genetic algorithm discovered a redundant term *u_x_u_xx_*, the coefficients of the true terms (*uu_x_* and *u_xxx_*) are accurate, which shows that the existence of the redundant term can compensate the error brought by the high noise and make the true terms accurate. This is why the generalized genetic algorithm can guarantee the correct terms to be included under high noise levels.

**Table S2.** Comparison between the true PDE and the outcomes from the generalized genetic algorithm, direct least squares regression on the true terms, and the PIC.

| The true PDE | $u_{t}=-uu_{x}-0.0025u_{xxx},$ |
| --- | --- |
| Direct least squares regression  on the true terms | $u_{t}=-0.490uu_{x}-0.0011u_{xxx}$ |
| The discovered PDE by  the generalized genetic algorithm | $u_{t}=-0.930uu_{x}-0.0027u_{xxx}-0.0015u_{x}u_{xx}$ |
| Ultimate discovered PDE by PIC | $u_{t}=-1.043uu_{x}-0.0026u_{xxx}$ |

**3.3 The analysis of noise tolerance for PIC**

In this work, the PIC has been proven to be able to discover parsimonious forms of governing equations from discrete data with high levels of noise. In some cases, it can be robust to up to 200% noise. The PIC method’s ability to tolerate high levels of noise is mainly due to three key factors: noise smoothing of the surrogate model; the calculating process of *p*-loss; and the calculating process of *r*-loss. While prior works have shown that neural networks are effective at eliminating the influence of data noise, they have limited noise-smoothing ability when faced with high levels of noise. An example is provided in Fig. S4, where the neural network is trained from 10,000 data selected from the solution of the KdV equation with 100% Gaussian noise. The constructed surrogate model exhibits apparent deviation, which can result in larger errors in the calculated derivatives. As a result, it is difficult to identify the correct PDE under high levels of noise.

Our proposed PIC manages to solve this problem by constructing the PINN surrogate models to further eliminate the noise and identify the correct PDE from candidates. In the PIC, some potential combinations are determined by calculating the *r*-loss. These potential combinations are utilized to be the physical constraint to construct new surrogate models based on the trained neural networks. As shown in Fig. S4, the trained PINN with the correct PDE is much more accurate; whereas, the trained PINN with the wrong PDE increases the error. This means that the process of calculating *p*-loss not only facilitates distinguishing the correct PDE, but further reduces the data noise, as well.


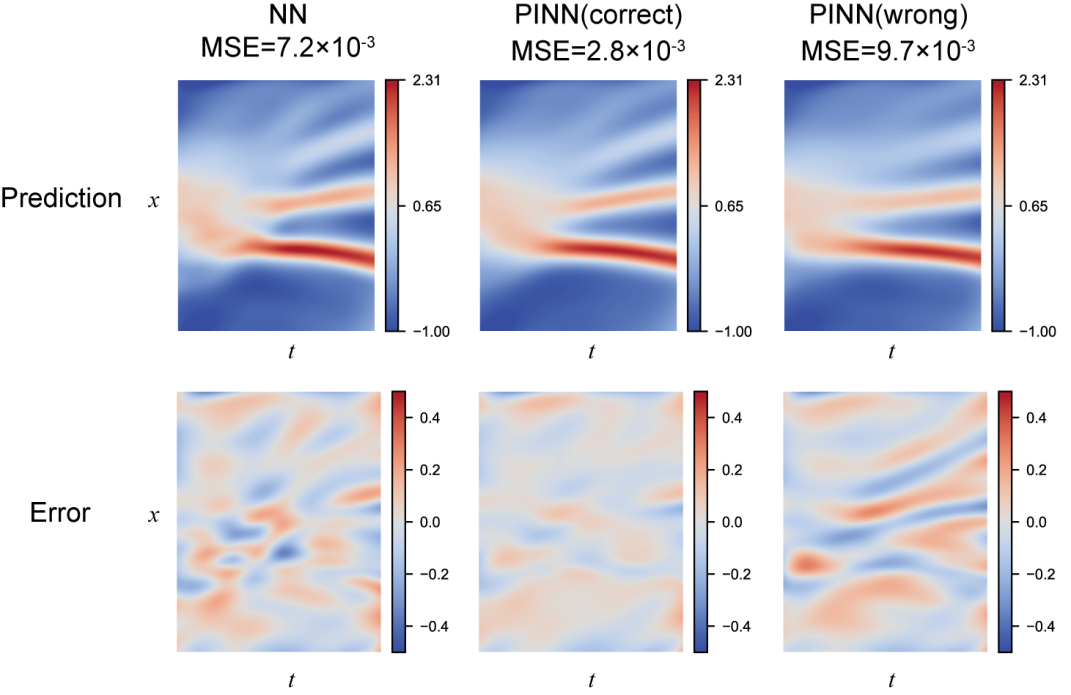


**Fig. S4. The performance of different surrogate models.** The left is the neural network trained by the noise data. The middle is the trained PINN with the correct PDE when calculating *p*-loss. The right is the trained PINN with the wrong PDE when calculating *p*-loss.

Meanwhile, it is revealed that some redundant terms may be identified to compensate for the error brought by high levels of noise. Therefore, the moving horizon technique is utilized to calculate *r*-loss and measure the parsimony of the discovered PDE to distinguish the redundant terms. In order to better demonstrate the function of the moving horizon technique, we visualize the variation of coefficients in the moving horizon in Fig. S5. Here, three types of combinations are illustrated, including: the true PDE (Fig. S5a); the false PDE (Fig. S5b); and the true PDE with redundant terms (Fig. S5c). It is evident that the mean *cv* of the true PDE is the smallest, while the mean *cv* is much larger for the true PDE with redundant terms. The main reason for this is the high variance (*cv*=0.144) from the redundant term *uu_xxx_*. This proves the validity of the moving horizon technique to distinguish the redundant terms, which allows the *r*-loss to measure the parsimony of the discovered PDE.


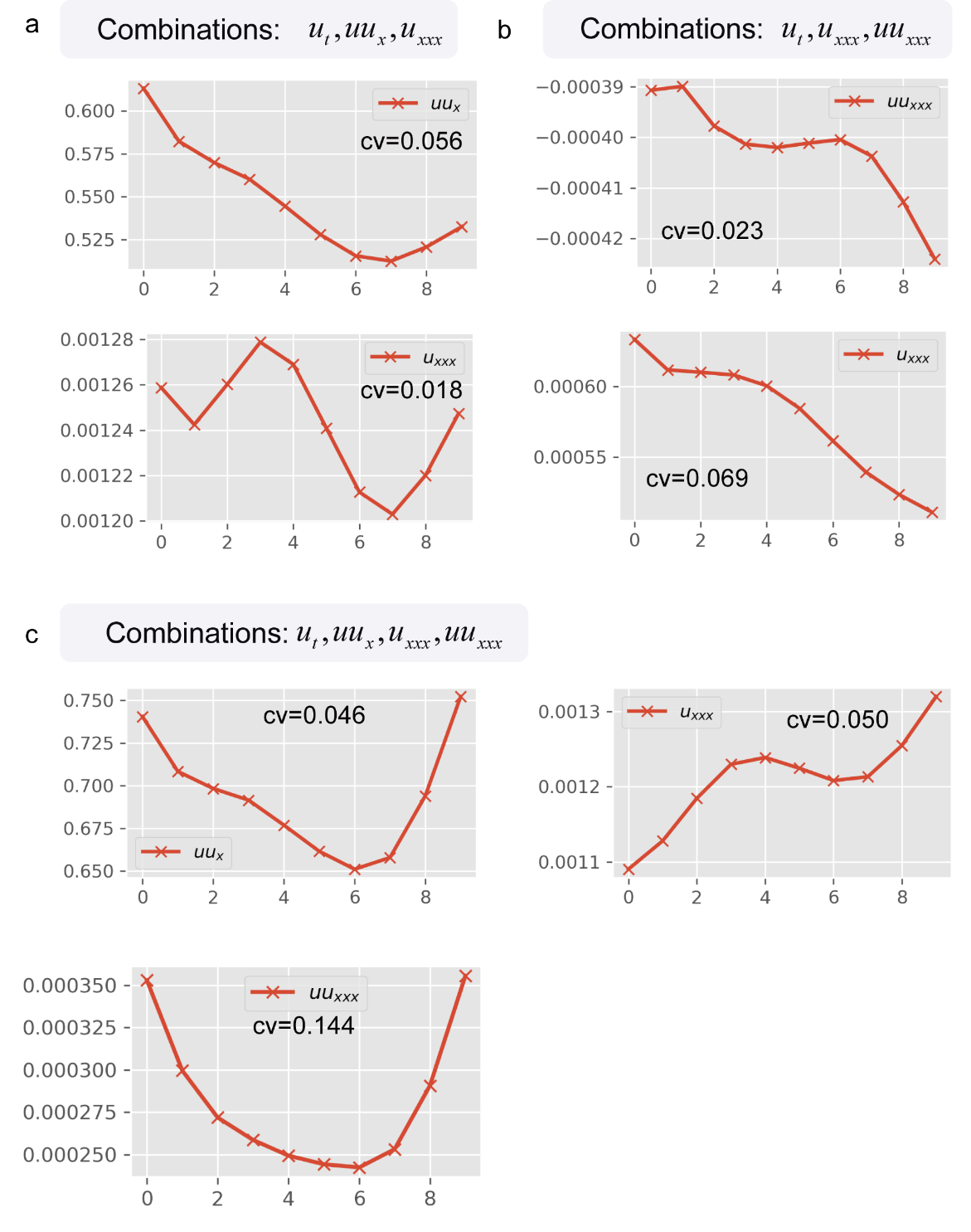


**Fig. S5. The variation of coefficients in the moving horizon for different combinations.** **a,** The true PDE. **b,** The false PDE. **c,** The true PDE with redundant terms. The KdV equation under 100% Gaussian noise is taken as an example here. The *x*-axis represents different horizons, and the *y*-axis represents corresponding coefficients.

**3.4 Maximum noise robustness of PIC**

In the manuscript, seven canonical PDEs are employed for proof-of-concept. In order to explore the robustness of the PIC to high noise, we increase the noise level by 25% until the correct PDE form fails to be discovered. The maximum noise robustness and corresponding discovered equations are displayed in Table S3.

**Table S3.** The result of the PIC when identifying seven canonical PDEs from data with high levels of noise. The noise level is the maximum noise where the correct PDE form can be discovered, and the activation function is the better one when identifying each PDE. The utilized data sizes are 10,000 for all cases.

| **Equation name** | **Equation form** | **Activation function** | **Noise level** | **Discovered equation** |
| --- | --- | --- | --- | --- |
| KdV equation | $u_{t}=-uu_{x}-0.0025u_{xxx}$ | Sin | 100% | $u_{t}=-1.043uu_{x}-0.0026u_{xxx}$ |
| Burgers equation | $u_{t}=-uu_{x}+0.1u_{xx}$ | Rational | 75% | $u_{t}=-0.960uu_{x}+0.078u_{xx}$ |
| Convection diffusion equation | $u_{t}=-u_{x}+0.25u_{xx}$ | Rational | 200% | $u_{t}=-1.02u_{x}+0.251u_{xx}$ |
| Chaffee-Infante equation | $u_{t}=u_{xx}-u+u^{3}$ | Rational | 50% | $u_{t}=1.108u_{xx}-1.312u+1.117u^{3}$ |
| Allen-Cahn equation | $u_{t}=0.003u_{xx}+u-u^{3}$ | Rational | 50% | $u_{t}=0.003u_{xx}+0.970u-1.007u^{3}$ |
| Wave equation | $u_{tt}=u_{xx}$ | Sin | 175% | $u_{tt}=0.976u_{xx}$ |
| KG equation | $u_{tt}=0.5u_{xx}-5u$ | Rational | 200% | $u_{tt}=0.465u_{xx}-5.55u$ |

**3.5 Comparison with existing methods**

In this section, the comparison between PIC and the state-of-the-art (SOTA) method in present literature for discovering seven canonical PDEs with 10,000 data is conducted, and the result is presented in Table S4.

**Table S4.** Comparison between the PIC and the SOTA method in the present literature for discovering seven canonical PDEs with 10,000 data. The results are provided in the form of maximum noise level (relative coefficient error). For the SOTA method, superscripts 1, 2, and 3 refer to the PDE-READ method [18], the R-DLGA method [19], and the DLGA method [5], respectively. The best results are shown in bold.

| **Equation name** | **SOTA method** | **PIC** |
| --- | --- | --- |
| KdV equation | 100% (64.5%)^1^ | **100% (4.15%)** |
| Burgers equation | **100% (10.0%)^1^** | 75% (13.0%) |
| Convection-diffusion equation | 100% (5.15%)^2^ | **200% (1.20%)** |
| Chaffee-Infante equation | 15% (1.46%)^3^ | **50% (17.9%)** |
| Allen-Cahn equation | **100% (72.5%)^1^** | 50% (1.23%) |
| Wave equation | 100% (1.20%)^2^ | **175% (2.40%)** |
| KG equation | 100% (6.10%)^1^ | **200% (9.0%)** |

**Table S5.** The discovered PDE by the PIC for the parametric convection-diffusion equation.

| **Noise level** | **Discovered equation** |
| --- | --- |
| 0% | $u_{t}=-(0.999+0.252\sin(1.00x))u_{x}+0.995u_{xx}$ |
| 25% | $u_{t}=-(0.983+0.228\sin(0.99x))u_{x}+0.996u_{xx}$ |
| 50% | $u_{t}=-(1.012+0.298\sin(1.059x))u_{x}+0.992u_{xx}$ |
| 75% | $\begin{matrix} u_{t}=-(1.17e^{-0.034x}+0.269\cos(-1.37x))u_{x} \\ +0.919xe^{-0.312x}u_{xx} \end{matrix}$ |

**3.6 Discovery of the Navier-Stokes equation**

In this study, the PIC method has successfully identified the 2D Burgers equation from sparse data and high levels of noise, demonstrating its stability for 2D problems. To further evaluate the method’s performance, a more challenging 2D system, known as the Navier-Stokes equation, is investigated. This equation describes viscous incompressible fluid flow, such as lid-driven cavity flow, and can be written as follows:

. (S.10)

Eq. (S.10) can be transformed into:

$u_{t}+v_{t}=-(uu_{x}+vu_{y}+uv_{x}+vv_{y})-(p_{x}+p_{y})+0.01(u_{xx}+u_{yy}+v_{xx}+v_{yy})$. (S.11)

The dataset is generated from simulation where $x\in(0,1), y\in(0,1)$, and $t\in[0,1]$. The number of *x*, *y*, and *t* is 98, 98, and 100, respectively. Therefore, there are 960,400 data in the dataset. Among them, 400,000 (41.6%) data are randomly selected to train the neural network. The meta-data are the grid data of 20 spatial observation points in the domain$x\in[0.1,0.9]$, 20 spatial observation points in the domain$y\in[0.1,0.9]$, and 20 temporal observation points in the domain $t\in[0.05,0.95]$. The meta-data size is 8,000. For the neural network, the inputs are *x*, *y*, and *t*, and the outputs are *u*, *v*, and *p*. For the genetic algorithm, the population size, iteration, and *l0*_penalty are 4,000, 400, and 0.001, respectively. The results of the PIC method applied to data containing different levels of noise are summarized in Table S6. These results demonstrate that the PIC method can accurately discover the correct PDE even when faced with up to 50% noise and discrete data. Furthermore, the error of optimized coefficients remains relatively low, especially for the advective acceleration term, with a relative error of approximately 5%. For higher levels of noise, the discovered PDE may differ from the reference, but most of the correct terms are still discovered. These results suggest that the PIC method has the potential to handle more challenging problems with more complex terms and complicated dynamic systems.

**Table S6.** The discovered PDE by the PIC for the Navier-Stokes equation.

| **Noise level** | **Discovered equation** |
| --- | --- |
| 0% | $\begin{matrix} u_{t}+v_{t}=-1.029(uu_{x}+uv_{x})-1.052(vu_{y}+vv_{y})-0.987(p_{x}+p_{y}) \\ +0.011(u_{xx}+u_{yy})+0.011(v_{xx}+v_{yy}) \end{matrix}$ |
| 25% | $\begin{matrix} u_{t}+v_{t}=-1.039(uu_{x}+uv_{x})-1.021(vu_{y}+vv_{y})-0.967(p_{x}+p_{y}) \\ +0.011(u_{xx}+u_{yy})+0.010(v_{xx}+v_{yy}) \end{matrix}$ |
| 50% | $\begin{matrix} u_{t}+v_{t}=-1.011(uu_{x}+uv_{x})-1.074(vu_{y}+vv_{y})-0.988(p_{x}+p_{y}) \\ +0.011(u_{xx}+u_{yy})+0.010(v_{xx}+v_{yy}) \end{matrix}$ |
| 75% | $\begin{matrix} u_{t}+v_{t}=-1.194(vu_{y}+vv_{y})-1.100(p_{x}+p_{y}) \\ +0.013(u_{xx}+u_{yy})+0.008(v_{xx}+v_{yy}) \end{matrix}$ |
